# Modeling the Effect of Disorder in the Two-Dimensional Electronic Spectroscopy of Poly-3-hexyltiophene in an Organic Photovoltaic Blend: A Combined Quantum/Classical Approach

**DOI:** 10.1021/acs.jpcc.3c01080

**Published:** 2023-03-15

**Authors:** Elisa Palacino-González, Thomas L. C. Jansen

**Affiliations:** Zernike Institute for Advanced Materials, University of Groningen, 9747 AG Groningen, The Netherlands

## Abstract

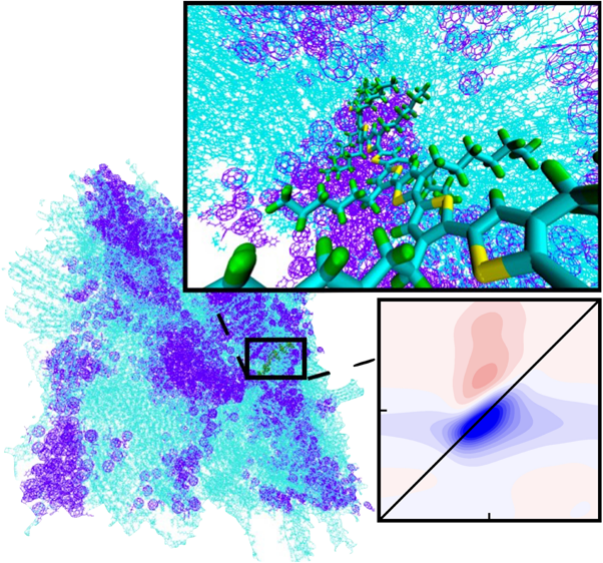

We introduce a first-principles model of the 12-mer poly-3-hexyltiophene (P3HT) polymer system in the realistic description of an organic photovoltaic blend environment. We combine Molecular Dynamics (MD) simulations of a thin-film blend of P3HT and phenyl-C61-butyric acid methyl ester (PCBM) to model the interactions with a fluctuating environment with Time-Dependent Density Functional Theory (TDDFT) calculations to parametrize the effect of the torsional flexibility in the polymer and construct an exciton-type Hamiltonian that describes the photoexcitation of the polymer. This allows us to reveal the presence of different flexibility patterns governed by the torsional angles along the polymer chain which, in the interacting fluctuating environment, control the broadening of the spectral observables. We identify the origin of the homogeneous and inhomogeneous line shape of the simulated optical signals. This is paramount to decipher the spectroscopic nature of the ultrafast electron-transfer process occurring in organic photovoltaic (OPV) materials.

## Introduction

The P3HT:PCBM blend is a OPV bulk heterojunction material that has been extensively studied and nowadays serves as a prototypical system to investigate the ultrafast charge-transfer process involved in photovoltaic applications.^1−6^ Most of the first-principles studies performed on these systems have been done in vacuo,^7−9^ where the connection between the structural disorder and the material optical properties is not explicitly included in the theoretical modeling which is, in this sense, still much underdeveloped. Nevertheless, the effect of the environment-induced disorder on the optical properties of these materials has been demonstrated to be crucial for the design of new organic photovoltaic materials.^10−14^ In this work, we fill this gap by introducing a first-principles model describing the optical properties of the electron-donor P3HT system, which includes the effect of the disorder induced by a realistic description of the OPV blend. Our description allows reproducing the main features of the experimental spectra available on P3HT thin-films.

Recent measurements of time-resolved optical spectra and quantum dynamical simulations on P3HT:PCBM thin-films revealed the presence of a strong vibronic coupling^15,16^ at the heterojunction between the two species that governs the ultrafast charge separation. As has been addressed by previous work on these systems,^17−19^ the efficiency of this process strongly depends on the morphology and optical properties of the surrounding blend environment. Recent experiments performed on P3HT thin-films^20^ support the generally accepted picture that a broad distribution of chromophores of different spatial extensions arises from the torsional disorder and confinement due to defects in the polymer chains. Indeed, extensive theoretical studies on the photophysics of conjugated oligomers analogous to the P3HT system^21−24^ address an existing correlation between the shape of the absorption spectra and the structural morphology of the electron-donor polymer. Along these lines, theoretical methods based on coarse-grained and atomistic MD simulations that predict blend morphologies have also been developed.^25^ Some authors have also demonstrated by combining DFT and TDDFT calculations with experimental data that the aggregation and morphology of bulk regioregular P3HT oligomers^26^ affect the optical properties and determine the efficiency of these materials. Furthermore, in the recent years, elaborate models^27−29^ involving multidimensional quantum dynamical strategies have been developed to describe the exciton delocalization process in oligothiophene systems after photoexcitation. Despite these efforts, a description of the effect of the environment on the optical and electronic properties of these systems is still missing.

In the present work, and with the prospect of developing a complete description of the charge-transfer process in prototypical OPV materials, we introduce a state-of-the-art modeling of a 12-mer P3HT electron donor system in a prototypical P3HT:PCBM thin-film blend which includes a realistic description of the fluctuating blend environment. We combine MD simulations of a thin-film blend for modeling the effect of the environment with dimer-based TDDFT calculations to parametrize the effect of the torsional potential between monomers in the chain. We introduce and analyze the role of ”kink” angles in the polymer chain, which determine the optical properties of the P3HT system in the blend environment. Our model allows the identification of the origin of the homogeneous and inhomogeneous broadening components from the torsional flexibility in the polymer chain and its interaction with the OPV blend. This study on a prototypical blend is critical to understand the effect of the environment on the light-induced dynamics preceding the charge separation in organic photovoltaic materials.

The paper is structured as follows. First the methodology and the model Hamiltonian describing the P3HT system in the P3HT:PCBM blend environment are presented. This is followed by the description of the main results, including a detailed configurational analysis of the P3HT chains present in the blend, as well as the effect of the disorder on the calculated optical spectra. After this, the simulated linear absorption and two-dimensional electronic spectra (2DES) are presented and analyzed, addressing the origin of the homogeneous and inhomogeneous line shape of the spectra. Finally, the results are briefly summarized and the main conclusions are presented.

## Methods

**Figure 1:**
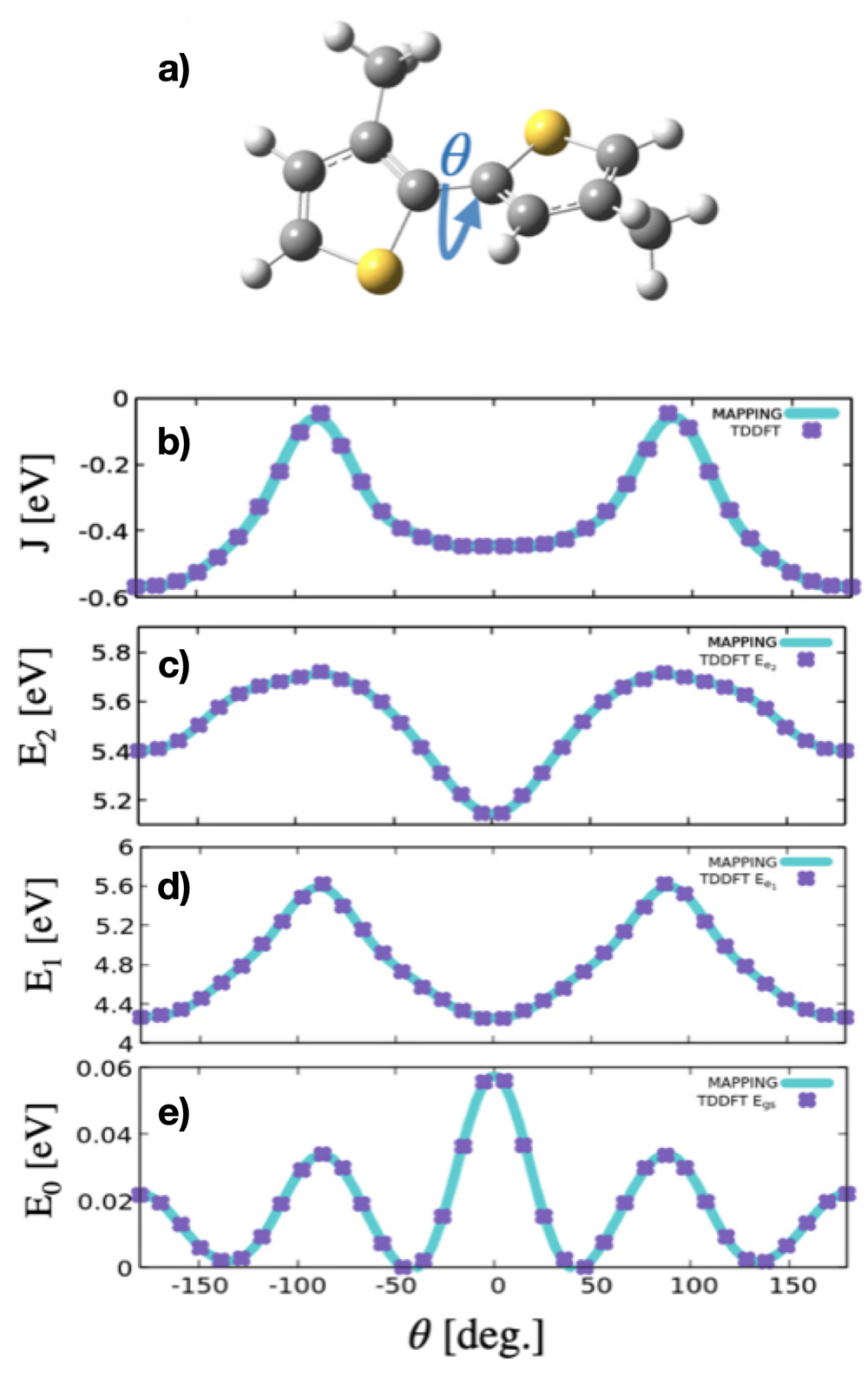
TDDFT calculated data for the dimer structure displayed in panel (a). Potential energy functions of the (e) ground, (d) first and (c) second excited electronic states and (b) excitonic coupling along the torsional angle defined by the atoms S–C–C–S.

A generalized Frenkel Hamiltonian^30^ in the nearest neighbor coupling approximation is adopted for modeling the photoexcitation in the P3HT, which for a general N-site basis is given by
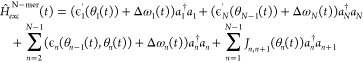
1ϵ_*n*_, θ_*n*_, and *J*_*n*,*n*+1_ are the excitonic energy of monomer *n*, the intermonomer torsional angles, and the excitonic coupling between sites *n* and *n* + 1, respectively. The torsional angle θ is depicted in Figure 1a for a P3HT dimer system. The terminal site energies, ϵ_1_^′^ and ϵ_*N*_^′^ only depend on one torsional angle. The key parameters are obtained from TDDFT calculations, as will be explained below. The approach of using a Frenkel exciton model based on the thiophene unit as a building block is supported by the transition density analysis included in Section S2 of the Supporting Information. We explicitly describe the interaction of the polymer with a surrounding blend consisting of 843 P3HT units and 1480 PCBM units by introducing the diagonal disorder *Δω*_*n*_(*t*). These fluctuations of the monomer site energies are defined by the blend environment shift, evaluated from MD simulations, by adopting an electrostatic *CHELPG* mapping scheme^31,32^ to calculate the electrostatic potential on the molecule induced by the fluctuating environment. In eq 1, the difference between end monomers (*n* = 1, 12) and central monomers (*n* = 2–11) is explicitly considered: while the energy of the end units is defined by a single torsional angle, the energy of the central units depends on the configuration of the two flanking angles connecting to the chain. Besides, this model treats each of the torsional angles in the chain independently. The purpose of the present model is to describe the electronic and optical properties of the P3HT system as affected by the environment defined by P3HT and PCBM domains in the blend. We neglect the presence of P3HT interchain coupling and its possible effects on the optical properties of the P3HT. The effect of this coupling in pure P3HT is known to result in a weak H-type coupling.^22^

Strong dependence between the optical and electronic structure–properties of oligothiophene systems and the conformational changes in the torsional angles between the thiophene rings in the chain have been reported in previous theoretical studies.^33^ Motivated by these studies showing a dominant role of the intrachain torsional angle on the optical properties of P3HT, we performed DFT and TDDFT calculations using the *ORCA* Quantum Chemistry Package^34^ to parametrize the excitonic Hamiltonian in eq 1. The dependence on the torsional angle defined by atoms S–C–C–S in the dimer system shown in Figure 1a was explicitly parametrized. In the parametrization, the hexyl substitutions of the P3HT system at positions 1 and 8 were replaced by methyl groups to reduce the computational cost. This strategy has successfully been adopted in the past,^7,26,35,36^ where it was demonstrated that the size of the alkyl substitution in the thiophene units does not have a significant effect on the electronic structure and optical properties of the P3HT. To parametrize the dependence on the torsional angle between monomers, a relaxed scan was performed on the dimer system. The profile of the potential energy functions for the ground (e), first (d), and second (c) electronic excited states along the torsional angle is shown in Figure 1. Based on previous work on similar oligothiophene systems^26,37−39^ we used the BHandHLYP functional and the def2-TZVP(-f)^40^ basis set to optimize the ground state geometry as well as for the TDDFT calculations. Single-point TDDFT calculations were also performed on a DFT-optimized geometry of the monomer system and are reported in Section S2 of the Supporting Information.

The excited state adiabatic energies obtained from the TDDFT calculations on the dimer were mapped into a 12-site diabatic Hamiltonian adopting a nearest-neighbor coupling approximation between adjacent thiophene units in the chain. The excitonic energies and couplings of the mapped diabatic Hamiltonian are defined by the two lowest excited state adiabatic energies *E*_1_ and *E*_2_, and are given by
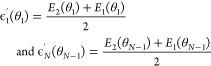
2for the terminal units (*n* = 1, *N*), and
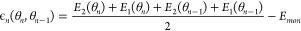
3for the central units (1 < *n* < *N* – 1). The excitation energy for the monomer, *E*_*mon*_ = 5.575 eV, obtained with the quantum chemistry calculations is used as well. Furthermore, the coupling between neighboring units were defined as
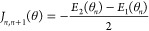
4Here, we used that for all dimer structures the lowest state had the highest oscillator strength reflecting a negative value of the coupling. This behavior reflects the *J*-aggregate character of the 12-mer P3HT system under study.

We evaluated the transition dipole moment vector for the 12 monomer units in the P3HT chain based on the oscillator strength data predicted for the monomer system by the TDDFT simulations. The orientation of the dipole moment vector for the methyl-substituted monomer system is reported in Figure S2a in Section S2 of the Supporting Information.

Panels b–e of Figure 1 summarize the main results from the DFT and TDDFT calculations on the dimer system. The most stable configuration presents a dihedral angle of ∼145°, corresponding to a quasi-planar configuration of the two thiophene rings. The profile of the excitonic coupling *J*(θ) evaluated from TDDFT calculations is shown in panel a. Purple dots represent the data retrieved by the TDDFT calculations, which was fitted to analytical functions of θ (turquoise solid lines). The electronic excitation from the ground to the first excited state presents the highest transition dipole moment which is characteristic of *J*-aggregates and is defined by excitonic coupling values < 0. The absolute value of the coupling in the dimer decreases as the dihedral angle between the two units deviate from the planarity, and it reaches a minimum value when the two monomers are perpendicular to each other (|θ| < 100°) Hereafter, we will refer from now on to this configuration as ”kink angle”, which breaks the delocalization in the conjugated polymer. For the planar configurations with θ = 0° (symmetric) and 180° (asymmetric), the oscillator strength of the transition from the electronic ground to the first excited state is the highest, characteristic of a *J*-aggregate configuration. As is shown in the excitonic coupling profile of Figure 1a, the coupling between the two monomers decreases as the units deviate from the planarity, and it reaches its lowest (absolute) value around |θ| = 90°.

With this mapping approach based on the dimer unit, we include a full independence of the *n* – 1 torsional angles in the chain from one another, which allows us to account for asymmetry in the polymer chain and the intramolecular conformational information defined by the dihedral angles. Furthermore, due to the different flexibility and chemical surrounding of the monomers, a distinction between the two monomers on the two ends of the chain (*n* = 1, 12) and the central monomers (*n* = 2–11) is included.

Due to the dynamics of the blend environment, the electronic and optical properties of the polymer experience an additional time-dependence. We account for this effect by incorporating a time-dependent term which describes the disorder of the excitonic energies (diagonal disorder), evaluated as a blend environment shift. For a given molecule of P3HT in the blend we calculate the blend environment shift experienced by each of the 12 monomers of the chain at each point of the trajectory. For an *N* × *N* diabatic Hamiltonian, the blend environment shift is evaluated for each monomer *n* by^32^
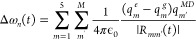
5where *q*_*m*_^*e*^ and *q*_*m*_^*g*^ denote excited state and ground charges on atom *m* of unit *n. q*_*m*′_^*MD*^ is the MD force field charge of atom *m*′ in the solvent, and *R* is the distance between the atoms in the given unit and in the surrounding solution. The first summation in eq 5 runs over the *m* thiophene ring atoms, neglecting the charges from the aliphatic substitution and the hydrogen atoms, due to their small contribution. To account for the molecules in the environment contributing to the blend environment shift, a cutoff radius of *R*_*mm*′_ = 50 Å around the polymer molecule of study was used, where contributions to the energy disorder below 48.0 cm^–1^ were disregarded. The atomic charges in eq 5 were evaluated using the CHELPG calculation scheme,^31^ based on fitting atomic charges to reproduce the molecular electrostatic potential at a large number of points around the molecule. The calculated atomic charges are reported in Table S1 of Section S2 in the Supporting Information.

To validate our model we compare it with TDDFT single-point calculations on the 12-mer P3HT system. Details about the level of theory used and the structure of the DFT-optimized geometry of the 12-mer system are reported in Section S2 of the Supporting Information. The energies predicted by our model representing a dimer-based mapping on a 12-site Hamiltonian appeared to be 1.1 eV higher than the ones reported by the exact calculations on the full 12-mer system. We attribute the difference between the energy predicted by our model and the TDDFT data on the 12-mer system to the neglect of long-range interactions between nonadjacent monomers and interchain couplings in the model. This effect would be interesting to include explicitly in the future, but it would require parametrization beyond the dimer model. Indeed, as reported by previous work on similar oligomers,^26^ calculations on a minimal length of 5–10 monomers are required to describe the excitonic manifold correctly, using more multidimensional mapping protocols.^41^ To account in these terms for the limitations of our model, we shifted the adiabatic energies predicted by our model by the 1.1 eV difference evaluated on the first-principles calculations on the 12-mer system. This shift is purely evaluated from TDDFT calculations and it allows to match the experimental absorption spectra measured on P3HT thin-films.

The DFT calculations predicted an optimized configuration for the 12-mer P3HT system defined by an average dihedral angle of θ ∼ 165–175°, in agreement with the data at room temperature reported by the theoretical studies of Simine et al.^14^ and with the MD simulations used here.^25^ This value differs from the data shown in Figure 1e corresponding to the DFT calculations on the dimer system, which can be attributed to steric effects due to the different length in the polymer. To compare the optimal torsional angle in different chain lengths, additional DFT/TDDFT simulations were performed on a 3-mer P3HT structure, which predicted an optimized dihedral angle of ∼115–123°, in contrast to the 130° value obtained for the dimer system. The data of the 3-mer system and a comparison with the one from our dimer model is included in Section S2 of the Supporting Information. Despite our dimer-based model differs in the dihedral angle with the 12-mer system, it predicts very well the adiabatic energies when extending to longer chains, independently on the dihedral values, as it is shown by the comparison with the TDDFT data on a 3-mer and 12-mer structure (see Table S5 in the Supporting Information). Additional calculations on the effect of the chain length on the electronic and optical properties of the P3HT, and the correction factors in the adiabatic energies predicted by our model are reported in Table S4 of the Supporting Information.

To model the environment, we simulated a MD trajectory emulating the experimental conditions of a thin-film organic photovoltaic blend. A simulation box representing a regioregular mixture of P3HT:PCBM of dimensions 29.6 × 29.6 × 4.5 nm containing 844 units of P3HT of the same length (12-mer) and 1480 units of PCBM was prepared based on previous work.^25^ A production run of 12.5 ps was calculated using a d*t* = 2 fs time step. The reference temperature of 298.15 K and pressure of 1.0 bar were used in the production phase (see Section S1 of the Supporting Information for additional details on the MD simulations).

The configurations generated by the MD simulations were used to construct Hamiltonian trajectories for the system as discussed above. We simulate the linear and third-order responses with the *NISE* program utilizing the Numerical Integration of the Schrödinger Equation (NISE) approach.^42,43^ The system Hamiltonian trajectory defined by eq 1 were used to calculate spectra for independent polymers. The dipole moment trajectory is derived from the MD data and contains the Cartesian coordinates of the transition dipole moment of the first electronic excitation for each of the site monomers in the P3HT chain. Each trajectory contained 3125 snapshots and spectra were calculated for starting configurations with every 100 snapshot. The time step in the propagation is 4 fs. The coherence times were varied from 0 to 256 fs. We used the parallel polarization two-dimensional electronic spectra for different waiting times. The NISE method does not account for thermal relaxation and potential self-trapping, however, the method has been shown to give accurate absorption spectra and two-dimensional electronic spectra for short waiting times, where relaxation is of minor importance.^44^

## Results and Discussion

**Figure 2:**
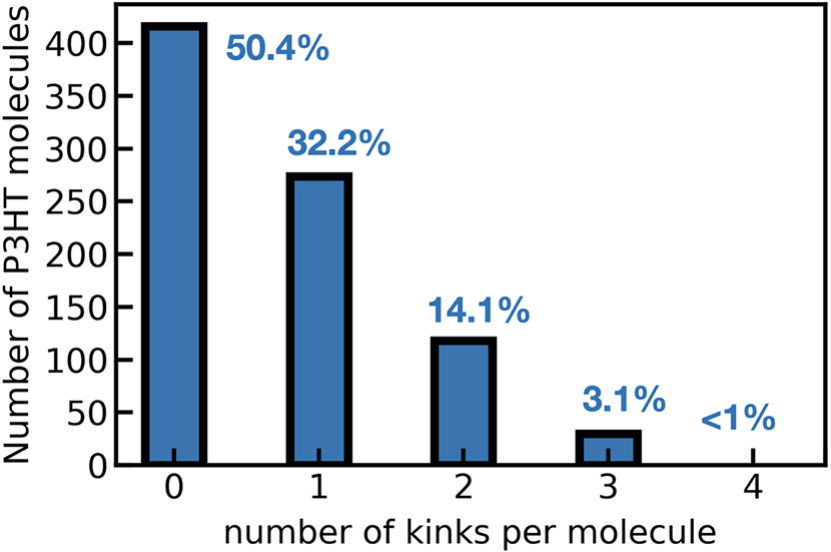
Statistical analysis of the presence of kink dihedrals in the P3HT units present in the blend along the MD trajectory for an averaged snapshot along the 12.5 ps trajectory.

It has been previously reported^45^ that changes in the morphology of the polymer due to its interaction with the blend environment affect the electronic and optical properties of the system. Therefore, we investigated the morphology of the polymer in the blend by analyzing the evolution of the different torsional angles along the MD trajectory for the 844 units of P3HT. We define the ”kink” angle between adjacent monomers as a specific configuration. Figure 2 shows the abundance of molecules in the blend with different number of kinks per chain, for an average snapshot along the 12.5 ps trajectory. Half of the molecules of P3HT in the blend do not have any kink along the trajectory. The second most abundant configuration contains one kink per chain (32.2% of the molecules), followed by a small fraction of molecules with two kinks (14.1% of the molecules). A small fraction of the polymers have more than two kinks and their contribution can be neglected. A further analysis of the trajectory revealed that kinks appears preferably at the first, middle and last positions of the P3HT chain (see Supporting Information for additional details on this).

**Figure 3:**
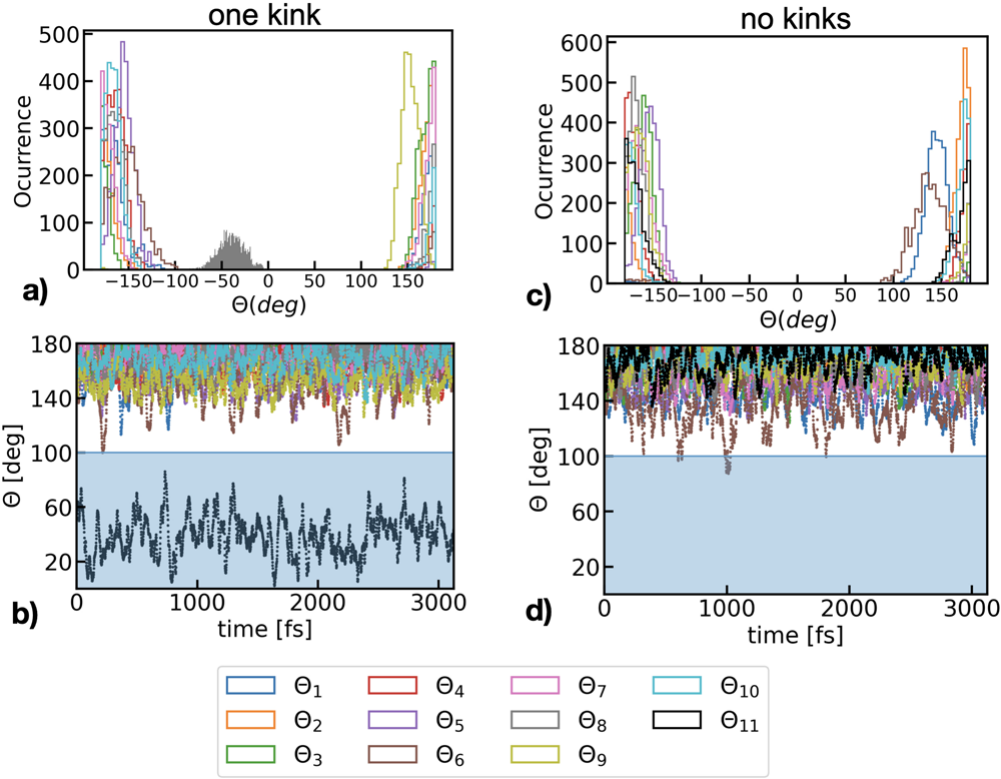
Conformational information along the MD trajectory for a selected molecule (id 417) of P3HT in the blend with one kink (upper panels) and no kinks (id 261) (lower panels). Panels (a,c) show the distribution of the 11 intramolecular torsional angles for each of the selected molecules. Panels (b,d) show the time-evolution of the dihedral angles.

Based on the analysis above we proceed to illustrate the effect of kinks in the optical properties of P3HT. We present the results from two representative examples from the ensemble of different P3HT molecules analyzed. We use a molecule with one kink (id 417) and one without kinks (id 261). The dihedral angle distribution and the evolution of the dihedral angles along the trajectory for the two molecules are displayed in Figure 3. The molecule with one kink, shown in panel a, adopts a quasi-planar configuration with a preferred dihedral value oscillating around the optimal geometry of θ = 140°. Negative values of the dihedral angles indicate a symmetric disposition of the rings where the two sulfur atoms are on the same side of the molecule. The presence of a kink is observed at the last position of the chain, and corresponds to a scenario where the monomers 11 and 12 are defined by almost-perpendicular planes. The analysis of the time-evolution of the kink revealed that it survives along the entire trajectory, as displayed in panel b. The data corresponding to the P3HT molecule with id 261 (see panel c), adopts a planar configuration close to the optimal geometry, with no kink angles in the chain. Due to the fluctuating environment, some molecules can experience substantial changes in its configuration which can be manifested with the emergence of kink angles in the chain. Panel d illustrates this effect, where a predominantly planar P3HT adopts a kinked configuration between monomers 6 and 7 for a few time frames along the trajectory. However, since this configuration is not statistically representative it is not displayed in the angles distribution shown in panel c. An analogous analysis for several P3HT molecules with different kink angles is contained in the Supporting Information.

**Figure 4:**
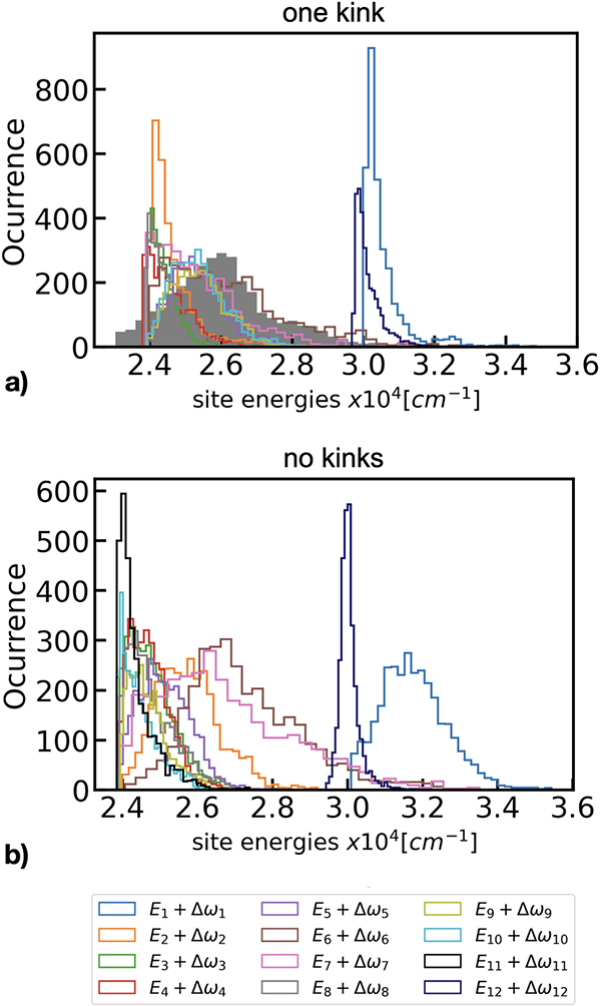
Distribution of monomer site energies along the MD trajectory for two P3HT molecules in the blend with (a) one kink (id 417) and (b) without kinks (id 261).

We further explore the effect of kinks and torsional flexibility in the chain originating from the interaction with the fluctuating environment on the electronic properties of the polymer. Figure 4 shows the distribution of the monomer site energies (considering the time-dependent blend environment shift) for two of the molecules in the blend with 1 kink and no kinks along the chain. We find that the broadening of the monomer site energies is dominated by the presence of the kink angles in the chain which induces a significantly broader distribution for the monomers connected by this special angle configuration. Furthermore, a clear spectral distinction for the central and end monomers is captured in our model. This is common in both molecules, with the energy of the latter appearing at higher frequencies due to the location in the chain in contrast to the red-shifted interchain monomers which are less mobile. In contrast, when the polymer has no kinks the site energies distribution is determined exclusively by the blend environment shift, broadening the energy of the central monomers similarly. It is relevant to mention that monomers at chain positions 6 and 7 in the molecule with no kinks appear to be particularly broader than the rest of central monomers, which can be justified by the time-evolution of the dihedral angles previously described in Figure 3d. This once more shows that the presence of kinks at any point along the trajectory dominates the broadening of the site energies.

To characterize further the effect of the kink we simulated trajectory-averaged density matrix maps of the group of P3HT molecules analyzed in the blend and can be found in the Supporting Information. We show that the presence of the kink determines the extent of the delocalization along the chain localizing the exciton based on the position of the kink on the chain. A similar analysis on the presence of kinks and its effect on the distribution of the excitonic couplings is reported and discussed in the Supporting Information.

**Figure 5:**
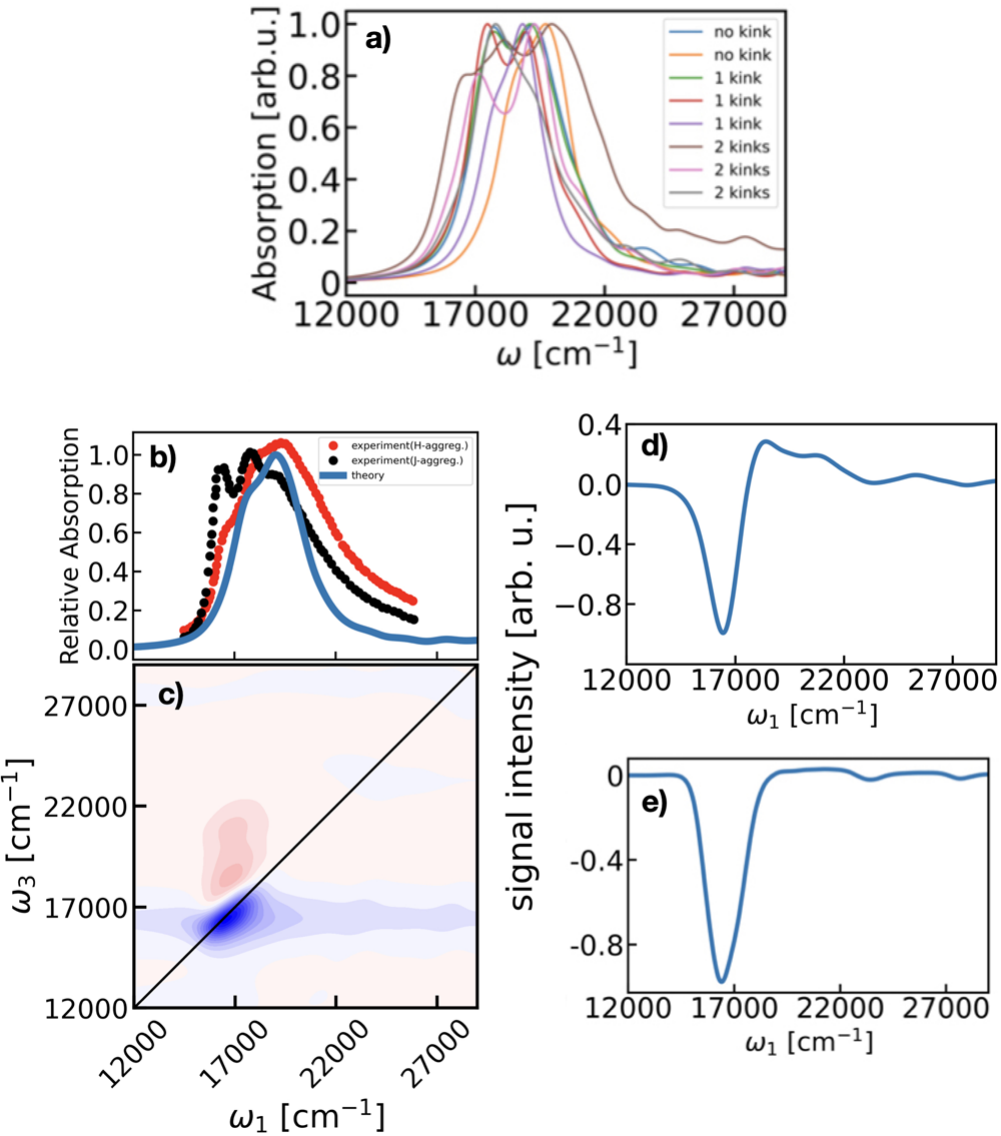
(a) Simulated linear absorption spectra of single molecules in the blend with different numbers of kinks. (b) Comparison of the (blue) ensemble-weighted average simulated spectra of P3HT in the P3HT:PCBM blend and measured linear absorption spectra of neat *H*-aggregate^46^ (red) and *J*-aggregate^46^ (black) P3HT thin-films. (c) Ensemble-weighted average simulated 2DES of P3HT at zero waiting time. (d) Vertical cut of the 2DES in panel (c) at excitation frequency of maximum absorption, ω_1_ = 1700 cm^–1^. (e) Diagonal cut of the 2DES in panel (c).

To test the validity of our model and explore the effect of the fluctuating blend environment on the optical properties of the P3HT, we simulate and analyze optical signals for a statistical representative ensemble of P3HT molecules in the blend. We performed simulations of the linear absorption and 2DES of single molecules at different positions in the blend to capture the heterogeneity of surrounding environments and presenting several number of kinks along the chain. To address the origin of the broadening to individual molecules and to the ensemble, we compare the spectra simulated for single P3HT molecules in the blend with the spectra evaluated on the ensemble of molecules. Data from the simulated ensemble-weighted averaged linear absorption and 2DES spectra is shown in Figure 5, where the weight averaging is derived from the analysis illustrated in Figure 2. Computational details of the signal calculation and the spectra from the single polymer are presented in the Supporting Information.

Figure 5a shows the simulated single polymer spectra in the blend with different number of kinks. Due to the different interacting environment (described by the blend environment shift) and the torsional flexibility (measured by the number of kinks) each polymer is subjected to, the central position and broadening of the spectra fluctuate slightly. Typical values for the single polymer broadening vary around 2600 cm^–1^. A few polymers containing more than 2 kinks show a broader spectra, but with a small contribution to the ensemble spectra of the blend. These differences coming from the single polymers translate in the ensemble-weighted averaged spectra as an inhomogeneous broadening originating from the ensemble averaging. Indeed, previous work by Thiessen et al.^20^ reports experimentally observed spectral shifts in the absorption spectra of single-chromophores originating from the different spatial extensions determined by the torsional disorder in the chains. This is in line with the experiments of Farouil et al.,^26^ the calculations in the same work performed on polymers of different lengths, as well as with our calculations reported in Table S4 of the Supporting Information, indicating that the DFT functional used in our model describes correctly this system. Our single-molecule signals capture the blue-shift of the spectra with the increasing number of kinks, however, this effect is not as pronounced as the one observed in the experiments^20^ on a thin-film of pure P3HT. We attribute this to the different environment in our simulations constituted by a mixture of PCBM and P3HT units, responsible for a more fluctuating environment affecting the kinks, therefore a strict comparison with the data from a pure P3HT sample should not be made. The neglect of disorder from intramolecular vibrations may also contribute to the difference. Moreover, our simulations describe P3HT units of the same length, therefore the additional blue-shift inherent to polymers of smaller size, which are reasonably present in the experiment, is not considered. Indeed, in Section S9 of the Supporting Information, we strengthen our interpretation by artificially removing the nearest-neighbor coupling between the two central monomers of a 12-mer molecule and recalculating the absorption spectrum. The resulting blue-shift is much smaller than the one obtained from an actual 6-mer system, showing that a kink in the chain does not make it optically equivalent to two shorter chains.

Figure 5b shows the ensemble weighted-average simulated linear absorption spectra (blue) and experimental spectra of a regioregular neat P3HT thin-film with *H*- and *J*- aggregate characters (red and black respectively).^46^ The simulated spectrum was calculated from the linear absorption spectra of an ensemble of P3HT molecules with different number of kinks. To render the final spectrum, a weighting factor was applied based on the number of kinks, following the statistical analysis in individual molecules presented in Figure 2. Our MD simulation box represents an thin-film of entangled 12-mer P3HT and PCBM units. In this case the polymer molecules interact weakly with one another, suggesting a *J*-character of the interchain coupling and finding a closer comparison with the experimental data shown in black. A fully faithful comparison of simulated spectra with the experimental data of the P3HT thin-film depicted in Figure 5b would require reproduction of the type of morphology and chain length distribution of the sample. In our model, we do not include interchain interactions; therefore, a detailed comparison with the experiment should be done with caution and our spectra should match the *J*-aggregate data with smaller interactions better.^46^ Despite this, the strongest absorption, centered at 18500 cm^–1^, and the main features of the experimental spectra are well-reproduced by our model. The intrinsic inhomogeneous broadening originated in the single-molecule signals from the different surrounding environment is hidden in the simulated ensemble-weighted averaged spectra due to the ensemble averaging, displaying a shoulder-like structure similar to the one observed in the experimental spectra of thin-films of pure P3HT.^26,46,47^ However, the vibronic progression feature (with 0–0, 0–1, 0–2 vibronic bands) observed in the experimental data of neat P3HT cannot be described by the present quantum-classical approach. This vibronic structure, which can affect the broadening of the spectra, is not included in the current model since it goes beyond the main purpose of this paper. The width of the simulated spectra measured at full-width and half-maximum (fwhm) of the spectra is ∼3000 cm^–1^, approximately 2400 cm^–1^ narrower than the one reported in the experiment. We attribute this difference to several sources: first, the inhomogeneity in the length of the P3HT chains in the experimental sample, in contrast to our simulation box where a fixed-length 12-mer P3HT system was considered. This distribution in the polymer length in the experiment translates into a broader spectral line shape. Additional data on the effect of the chain length on the simulated linear absorption spectra can be found on the Supporting Information. Second, to emulate the realistic environment of an organic photovoltaic material, our model includes a mixture of P3HT and PCBM units. This environment interacts with a given P3HT molecule differently to the one in a regioregular thin-film of pure P3HT, where the reported experimental spectra was measured. A comparison with the experiment has limitations as reconstructing all experimental parameters (such as the distribution of chain length) is challenging, if not impossible. Therefore, and due to these differences described above, a strict comparison of our model prediction with the experimental data presented in Figure 5b should be done with caution. Ongoing work is being performed to improve the current limitations of the current model. This includes adding a quantum mechanical description of the vibrational structure in the P3HT system to describe the vibronic character of the spectra, as well as a description of the interaction in between adjacent polymer molecules. Moreover, current developments are being performed toward extending the present model to include the interaction between non-neighboring monomers in the P3HT chain and account for contributions of charge transfer effects (see also Section 2 of the Supporting Information).^28,41^ These advancements will further improve the line shape of the simulated spectra allowing to obtain a predicted spectra exactly comparable to the available experimental data reported on neat P3HT thin-films.^26,46^

To address the origin of the homogeneous and inhomogeneous spectral broadening in the P3HT spectra and attribute them to the single-polymer and ensemble contributions we simulate and analyze 2DES signals, which represent the most complete characterization of the third-order electronic response of a system. Figure 5c shows the simulated ensemble-weighted averaged 2DES at zero waiting time. The spectrum displays two main features at the maximum absorption frequency of ∼17000 cm^–1^ corresponding to a combined ground-state bleach (GSB) and stimulated emission (SE) diagonal peak (negative blue) and excited-state absorption (ESA) cross-peak (positive red peak). The GSB and SE contributions overlap at the diagonal position of 17000 cm^–1^ and relate to the central peak position of the linear absorption spectra. A vertical cut at the maximum absorption peak of the 2DES in panel c is presented in panel d, from which an estimated homogeneous broadening of 1700 cm^–1^ is observed. A diagonal cut of the spectra was performed to extract the inhomogeneous and homogeneous contributions to the broadening, and it is shown in Figure 5e. The maximum intensity is red-shifted by ∼1500 cm^–1^ as compared to the linear absorption, as a consequence of the fourth-power dependence on the transition dipole moment, which enhances the intensity of the lowest energy excitonic level revealing the *J*-aggregate character of the P3HT system and reproduces nicely the experimental observation of the shift observed in P3HT blends.^47^ The red-shift we observe in the simulated spectra of P3HT in the P3HT:PCBM blend has a different nature to the one reported on recent single-molecule calculations on pure P3HT by Simine et al.^14^ In the latter, the red-shift is originating from an induced planarization in the higher-temperature state determined by the steric interactions of the side chains and the forces from the π-electrons on the nuclei. An inhomogeneous broadening of 1800 cm^–1^ was measured at fwhm of the spectrum, approximately twice the value of the inhomogeneous broadening extracted from analogous diagonal cuts on simulated single polymer 2DES (presented in Section S10 of the Supporting Information). This analysis reveals that the ensemble averaging accounts for almost half of the inhomogeneous broadening describing the P3HT excitation in the blend environment. An excited state absorption peak in the 2DES indicated by a spectral correlation between the excitation at 17000 cm^–1^ and the detection at 18500 cm^–1^ is also observed in the spectra. This is a signature of the exciton delocalization along the polymer chain.^48^ The comparison with the experimental 2DES of P3HT:PCBM blends^47^ is difficult because the experimental spectra present features of charge-transfer states due to the PCBM units, which are absent in the present model.

In a second step, we analyze 2DES simulated at increasing waiting times to address the time scale for the homogeneous dephasing, which suggests an approximate value of the electronic dephasing time of 100 fs, governing the excited-state dynamics of the P3HT due to the interaction with the fluctuating environment. The simulated spectra of the P3HT ensemble in the P3HT:PCBM blend for different waiting times is reported in Figure S28 of the Supporting Information. A decay constant of about 100 fs has also been observed in 2DES experiments on thin-films of P3HT,^47,49^ which also report a second decay constant in the picosecond time scale. Such decay constant is not observed in our simulations, suggesting a higher degree of disorder in the blend environment in contrast to the neat P3HT in which the experiments were performed. This reinforces our statement on the weak role of the kinks in shifting the single-chromophore absorption maxima in the blend in contrast to neat P3HT thin-films.

## Conclusion

In this study, we address the origin the spectral line shape of the P3HT system embedded in a P3HT:PCBM thin-film blend. We introduce a first-principles modeling of the 12-mer P3HT system based on TDDFT and MD simulations, which includes the explicit description of the blend environment defined by the bulk heterojunction of a mixture of P3HT and PCBM units. We use this to predict the linear absorption and 2DES spectra. A time-dependent configurational analysis of the individual P3HT molecules in the blend reveals the existence of torsional flexibility (kinks) in the polymer chain affecting the optical and electronic properties of P3HT. The predicted 2DES of the P3HT ensemble in the P3HT:PCBM blend attributes almost half of the inhomogeneous broadening to the averaging over the ensemble of P3HT molecules in the blend. For the individual P3HT molecules, the inhomogeneous broadening has two origins, the blend environment shift of the monomer site energies due to the fluctuating environment and the torsional flexibility of the polymer chains. The former one is crucial for planar configurations of the chain, whereas the latter dominates the site energies distribution of P3HT chains presenting kinks along the MD trajectory. We found the characteristic blue-shift in the single-molecule P3HT spectra due to the increasing number of kinks to be relatively low, because of the higher degree of disorder imposed by the blend environment in contrast to pure P3HT samples. From time-dependent calculated 2DES, we estimate a value of the dephasing rate for the excited state dynamics of P3HT of ∼100 fs, which has also been observed in 2DES experiments performed on P3HT thin-films.^47,49^ Last, the fragment-based electron–hole plots presented along the transition density analysis show a dominant Frenkel exciton character associated with the lowest-lying electronic states used in the parametrization, which strengthens our model. Some charge-transfer character is found in the higher-lying electronic states. A future improvement to the model would be to account for this charge-transfer character. Additional potential improvements to the current model include accounting for the quantum description of the vibrational structure of the P3HT system, including interchain couplings, as well as the effect of long-range interactions between nonadjacent monomers, which would improve the exact comparison with the available experimental data. Our presented model serves as a stepping stone for the development of a next-stage model that describes the photoinitiated quantum dynamics of the charge separation at the donor–acceptor heterojunction in prototypical organic OPV materials.

## Supporting Information Available

Details about the MD simulations, DFT-optimized geometries for the monomer, dimer, trimer, and 12-mer P3HT systems and TDDFT calculations, transition density plots, configurational analysis of the blend, data from excitonic couplings and energies and optical signals for different P3HT molecules in the blend (at varying *T*_pop_ waiting times), density matrix maps, and the data from the inverse participation ratio (PDF)
