# Supplementary material for: Modeling the Effect of Disorder in the Two-Dimensional Electronic Spectroscopy of Poly-3-hexyltiophene in an Organic Photovoltaic Blend: A Combined Quantum/Classical Approach
Source: J Phys Chem C Nanomater Interfaces. 2023 Mar 15;127(14):6793–801. doi: 10.1021/acs.jpcc.3c01080 (PMC10108354; doi:10.1021/acs.jpcc.3c01080)
Supplement: Supplementary file 1 — jp3c01080_si_001.pdf [file jp3c01080_si_001.pdf]

# Supporting Information: Modelling the Effect of Disorder in the Two-Dimensional Electronic Spectroscopy of Poly-3-hexyltiophene in an Organic Photovoltaic Blend: a Combined Quantum/Classical Approach

Elisa Palacino-González* and Thomas L. C. Jansen

*Zernike Institute for Advanced Materials, University of Groningen, 9747 AG Groningen, The Netherlands*

E-mail: e.palacino.gonzalez@rug.nl

# S1 Molecular Dynamics details

In Figure S1, a top view on the final equilibrated morphology of a P3HT:PCBM thin-film blend on which a 12.5 ps Molecular Dynamics (MD) trajectory was simulated is displayed. The initial input geometries of the system in the simulation box prior to the equilibration phases and details of the simulation protocol have been adopted from the work of R. Alessandri *et al.*[1] The P3HT:PCBM weight ratio is 0.7:1.0 containing 844 units of P3HT and 1480 units of PCBM.

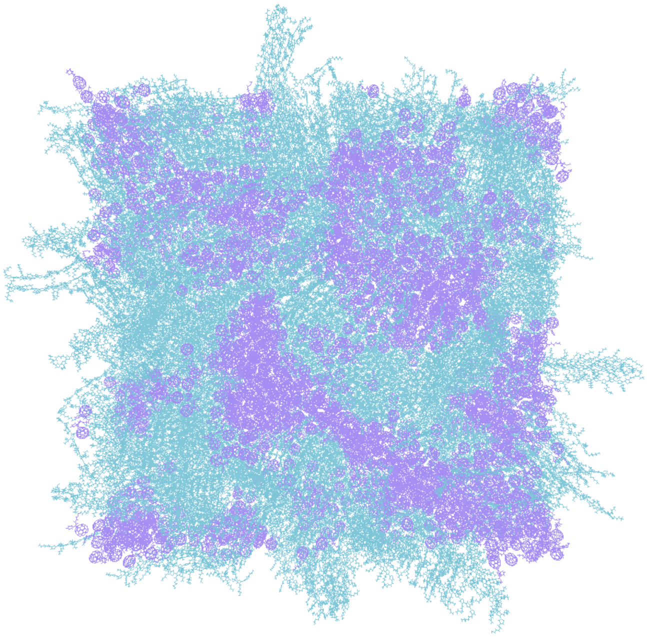

Figure S1: Top view of the P3HT:PCBM thin-film blend morphology of simulation box size 40 x 40 x $\sim$ 5 $nm^3$.

# S2 Quantum Chemistry calculations

We used density functional theory (DFT) for ground-state geometry optimizations. Figure S2 shows the optimized structures of the monomer, dimer and 12-mer systems. The hexyl substitutions at positions 3 and 8 have been replaced by methyl groups for reducing the com-

putational costs, since the substitution does not produce significant changes in the electronic structure and optical properties. Figure 2 a) shows the orientation of the dipole moment evaluated on the monomer system, with a labeling on the ring atoms. The direction of the transition dipole moment vector results from the sum along the ST1-CT2 and CT2-CT3 bonds in the tiophene ring. The dimer system depicted in b) shows the torsional angle defined between the ST1-CT2-CT2'-ST1' atoms of the two monomer units. For the optimized geometry the dihedral angle is found to be 130°. A TDDFT scan along this torsional angle is performed and results discussed in the main paper. The DFT-optimized structure of the 12-mer system present in the MD blend is displayed in c), where a single point excited-state calculation was performed to evaluate the excitation energies on the full-length system for reference. The dihedral angles for the optimized geometry are shown in Table S1. The calculations for the three systems were performed at the BHandHLYP/def2-TZVP(-f) level of theory.[2] All quantum-chemical calculations including the evlauation of the transtion density plots from Figure S4 were performed using the *ORCA* quantum chemistry software.

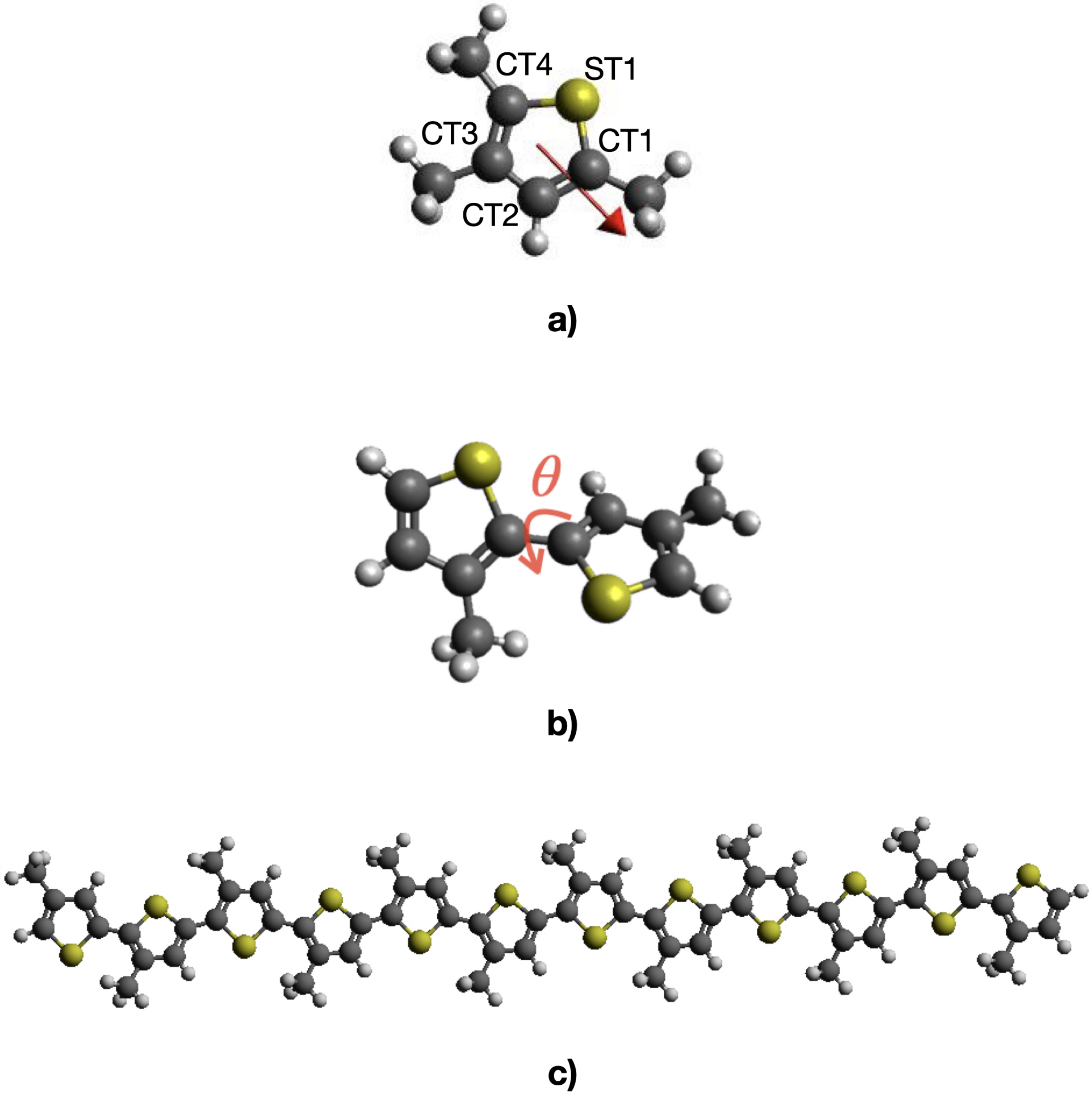


Figure S2: DFT-optimized ground state geometry of the a) monomer, b) dimer and c) 12-mer P3HT structures with methyl substitutions.

Figure S3 shows the DFT-optimized geometry of a 3-mer P3HT system. The optimized dihedral angles are found to be 152.7° and 154.2°.

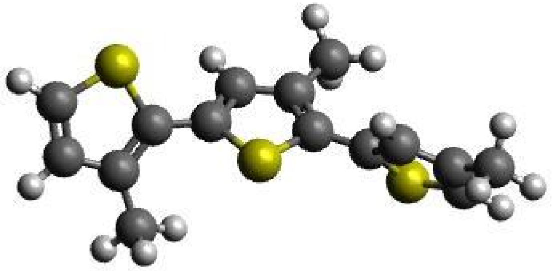

Figure S3: DFT-optimized ground state geometry of the 3-mer P3HT with methyl substitutions.

Table S1: Dihedral angles for the DFT-optimized geometry of the 12-mer system dsplayed in Figure S2 c).

| $\theta_1$ | $\theta_2$ | $\theta_3$ | $\theta_4$ | $\theta_5$ | $\theta_6$ | $\theta_7$ | $\theta_8$ | $\theta_9$ | $\theta_{10}$ | $\theta_{11}$ |
|---|---|---|---|---|---|---|---|---|---|---|
| 155.5° | -163.1° | 170.7° | -174.4° | -172.3° | 170.8° | -168.3° | 168.5° | -169.1° | -165.2° | 157.3° |

For the monomer system shown in Figure S2 a) we evaluated the CHELPG charges within the *ORCA* Quantum Chemistry Package[3] on the five ring atoms for the ground and the first-excited states, and are summarized in Table S2:

Table S2: DFT/TDDFT-evaluated atomic charges on the monomer atom rings for the electronic ground (left) and first-excited (right) states.

| Atom | Ground state [$e$] | Excited state [$e$] |
|---|---|---|
| ST1 | -0.108341 | -0.229621 |
| CT1 | 0.000215 | 0.060156 |
| CT2 | 0.284113 | 0.231265 |
| CT3 | 0.558783 | 0.566189 |
| CT4 | 0.179257 | 0.304573 |

In Table S3, we report the calculated TDDFT energies for the lowest lying adiabatic state of the dimer system for two different functionals. Our calculations show that the long-range-corrected $\omega$B97XD functional predicts a higher energy than the one predicted by the adopted BHandHLYP functional, blue-shifting the absorption spectra by 0.20 eV.

Table S3: TDDFT-evaluated adiabatic energies of the electronic transition with highest oscillator strength for two different functionals. A def2TZVP(-f) basis set was used in both calculations.

| BHandHLYP | $\omega$B97XD |
|---|---|
| 4.76 eV | 4.96 eV |

Last, Table S4 shows a comparison of the adiabatic energies predicted by our dimer model and the TDDFT calculations, for different chain lengths containing n units.

Table S4: Adiabatic energies of the lowest-lying electronic excited state for polymer systems with n units (first column). The second and third columns correspond to the data evaluated from the single-point TDDFT calculations on the DFT-optimized geometries and predicted by our model, respectively. The last column shows the energy difference between both approaches. The TDDFT calculations have been performed using the BHandHLYP functional and a def2TZVP(-f) basis set.

| n-mer | TDDFT [eV] | Dimer model [eV] | $\Delta$ [eV] |
|---|---|---|---|
| 1 | 5.41 | - | - |
| 2 | 4.76 | 4.73 | 0.03 |
| 3 | 3.52 | 3.93 | 0.41 |
| 4 | 3.10 | 3.66 | 0.56 |
| 6 | 2.52 | 3.23 | 0.71 |
| 8 | 2.39 | 3.24 | 0.85 |
| 12 | 2.18 | 3.18 | 1.0 |

Table S4 shows that our model predicts very well the adiabatic energy of the lowest-lying excited state of the 3-mer and 4-mer systems. When mapping the model on systems with higher number of units n, an increasing discrepancy with the TDDFT-predicted energies can be observed. For the 12-mer system of interest in this study, a correction factor of $\sim$ 1 eV in the adiabatic energy is required. The DFT-optimized structure of the systems considered

in Table S4 displayed different dihedrals values to the one presented in the dimer system. Although the dihedrals might play a role, this shift is largely due to electronic effects, and is originating from the absence of long-range interactions in the model. However, despite this limitation, our model is able to reproduce the red-shift experimentally observed[2] in the absorption spectra of P3HT when increasing the number of units in the chains. To conclude the analysis of the accuracy of our model, we report in Table S5 an additional comparison of the predicted energies and the TDDFT calculations for a trimer and a 12-mer system with typical dihedral angles directly taken from our MD simulations. For the trimer system, the dihedral angles were -174.2° and -48.4°.

From Table S5 it can be concluded that despite the value adopted by the dihedral angles in the P3HT chains, our model is able to reproduce the correction factor in the adiabatic energy, suggesting a purely electronic origin from the absence of long-range effects in our model, as described in the main text.

Table S5: Comparison of the energies from the single-point TDDFT calculations (second column) and model predictions (third column) on a 3-mer and a 12-mer system with dihedral geometries taken from the MD simulations. The column on the right shows the evaluated correction factors on the adiabatic energies derived from our model, which are almost identical to the ones reported on Table S4.

| n-mer | TDDFT [eV] | Dimer model [eV] | $\Delta$ [eV] |
|---|---|---|---|
| 3 | 3.59 | 4.00 | 0.41 |
| 12 | 2.23 | 3.18 | 0.95 |

Additionally, we analyze the Frenkel exciton character of the lowest electronic excited-state, responsible for the optical properties of the P3HT. Figure S4 shows the transition densities for the first excited-state of the a) dimer, b) trimer and c) 12-mer systems. A clear Frenkel exciton character is observed after excitation on the three systems, characterized by a delocalization of the electron density involving the $\pi$ orbitals along the polymer chain. This is a signature for the J-aggregate character of the P3HT.

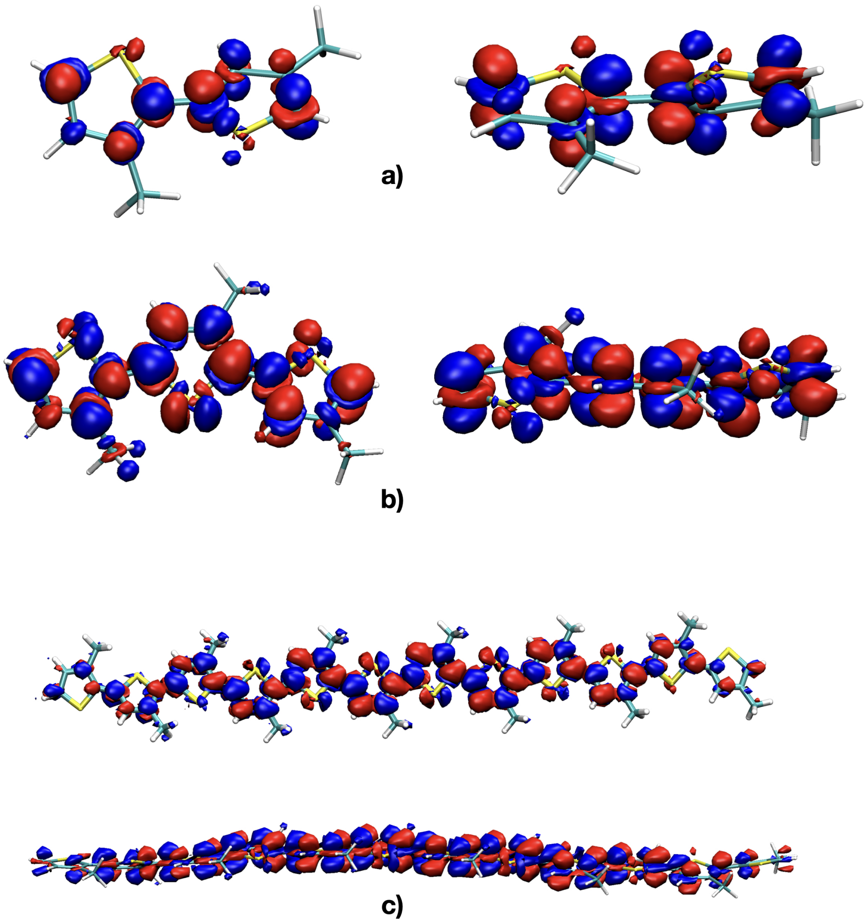


Figure S4: Transition density plots corresponding to the first electronic transition of the a) dimer, b) trimer and c) 12-mer systems. The single-point energy calculations have been performed on the corresponding DFT-optimized geometries shown in Figures S2 and S3.

To further describe the charge-transfer (CT) character associated to the excited states involved in our first-principles model, we perform a fragment-based transition density matrix analysis of the two lowest-lying excited electronic states (E1 and E2), for several dimer structures with different dihedral angles and the 12-mer. This analysis has been performed using the TheoDORE Computational package.[4] To understand the electron-hole distribution we plot the charge-transfer numbers in a two-dimensional plot, with the origin in the lower left corner (Figures S5 and S6 for the dimer and 12-mer structures, respectively). The fragments correspond to the monomer units in both structures (2 in the dimer and 12 in the

12-mer). The main diagonal represents the exciton contributions whereas the off-diagonal elements represent the charge-transfer.

Figure S5 displays the electron-hole correlation plots for the first (left) and second (right) excited electronic states of the dimer system used in our exciton model. Figure S5 shows the data corresponding to different dimer structures with fixed dihedral angles of a) 130° (DFT-optimized), b) 87°, c) 180°, d) 5.14°, e) 36°, and f) 150°. The analysis of these maps reveals a dominant localized exciton (LE) character for both electronic states, with a minor contribution from a CT character. Besides, the value of the dihedral angle does not play a significant role on the revealed nature of the states. A similar investigation has been reported for an analogous chromophore by previous authors.[5] Our analysis strengthens the validity of our current model, in which the two lowest-lying excited states of the dimer system were used to parametrize a Frenkel exciton Hamiltonian of the 12-mer P3HT system.

Figure S6 shows the electron-hole correlation plots for the DFT-optimized geometry of the 12-mer system. Left and right correspond to the data for the first (E1) and second (E2) electronic excited states, respectively. The nature of the E1 is dominated by a LE character, with the exciton spread over half of the fragments in the 12-mer chain. The map of E2 also reveals the excitonic nature of the state, which appears to be more localized on fragments along the chain. Both states present some CT character, however the analysis shows a dominantly LE character for both states. Further developments in our present model are planned to include a description of the charge-transfer character in the system Hamiltonian.

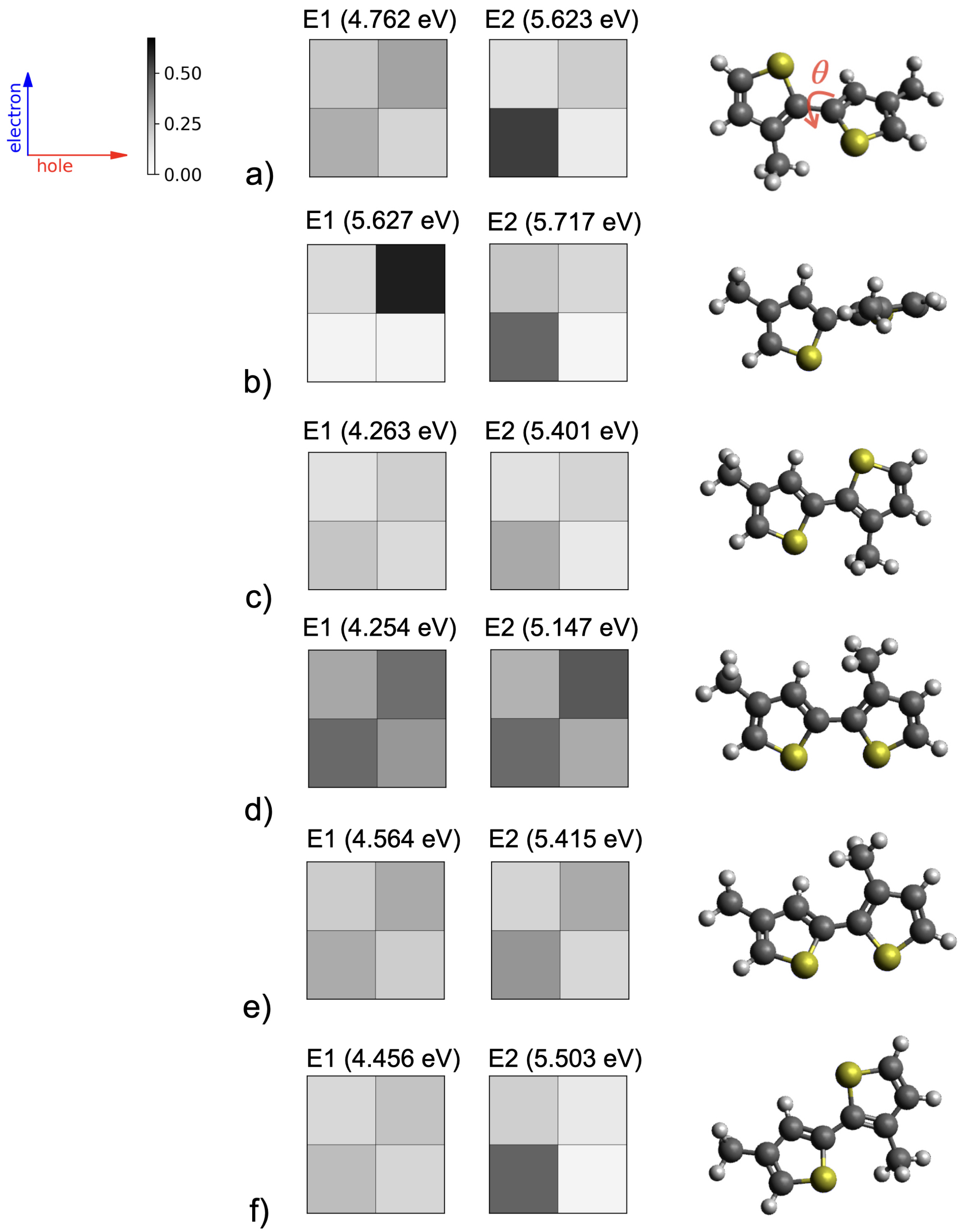


Figure S5: Electron-hole correlation plots of the dimer system used in the model parametrization for different dihedral angles: a) 130° (DFT-optimized), b) 87°, c) 180°, d) 5.14°, e) 36°, and f) 150°. E1 and E2 denote the first and second electronic excited states, respectively.

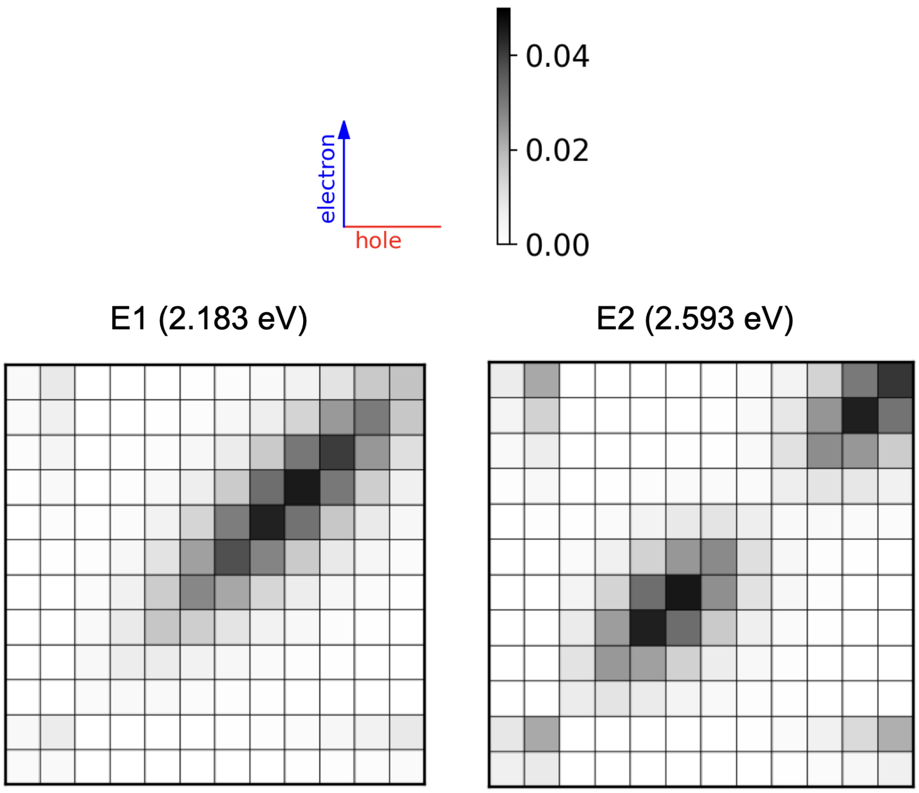


Figure S6: Electron-hole correlation plots of the DFT-optimized geometry of the 12-mer system used in our MD simulations. E1 and E2 denote the first and second electronic excited states, respectively.

# S3 Dihedral analysis configuration of single P3HT molecules

We analyzed the MD trajectory to extract the conformational information in the blend based on the number of kinks and their position in the P3HT chains. We selected several molecules in distinct positions in the blend which presented different number of kinks. Figure S7 shows the distribution of kinks along the chain for the 844 P3HT molecules present in the blend, for an average snapshot along the 12.5 ps trajectory. The distribution shows that the end (dihedral 11) and middle (dihedral 6) dihedrals are the preferred positions for the kink. Finding a kink at any other point along the chain is equally probable. From the data displayed in Figure 2 a) of the main text the second most abundant configuration of the molecules present in the blend have a single kink along the chain, which in accordance

with the analysis shown in Figure S7 will appear predominantly at on of the chain ending positions.

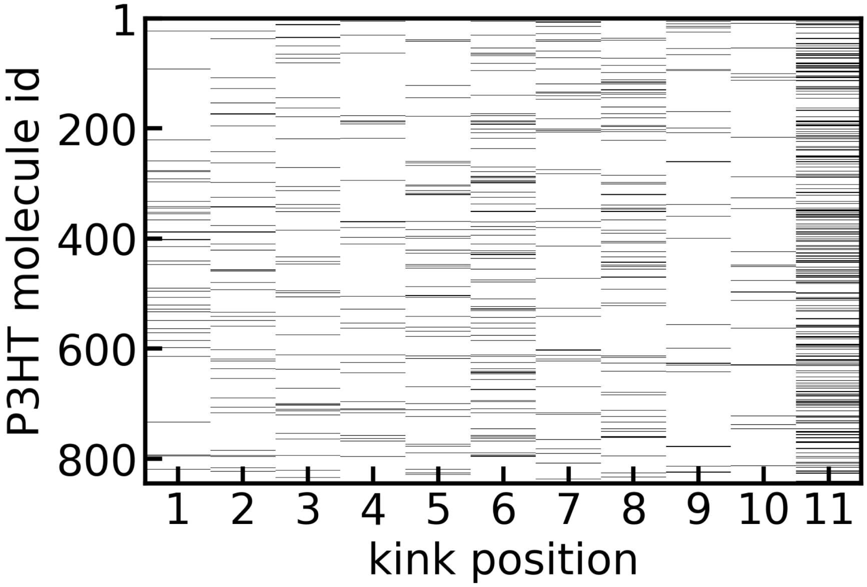


Figure S7: The statistical distribution of the position of kink angles along the chain for the 844 molecules of 12-mer P3HT in the blend for an average snapshot along the 12.5 ps trajectory. The $y-$axis is an index associated to a molecule of P3HT in the blend and $x-$axis is the position of the kink in the chain.

Figures S7, S8, and S9 show the distribution of dihedral angles along the MD trajectory for different molecules of P3HT in the blend without kinks, with one kink and two kinks, respectively. We selected molecules at random positions in the blend which presented different number of kinks along the chain. As seen in Figure S8, molecules without kinks adopt an average configuration along the trajectory, close to the planarity, with angles fluctuating around the minimal energy configuration defined by dihedral angles of $\theta \sim 145°$. Figure S9 shows the data corresponding to three different molecules of P3HT presenting a kink at position a) and b) 11 and c) 5. Figure S10 shows analogous data for two molecules presenting two kinks at positions a) and b) (3,11) and one molecule with a kink at positions c) (2, 7). The kink is defined by dihedrals values in the range given by $\theta \sim |0° - 100°|$.

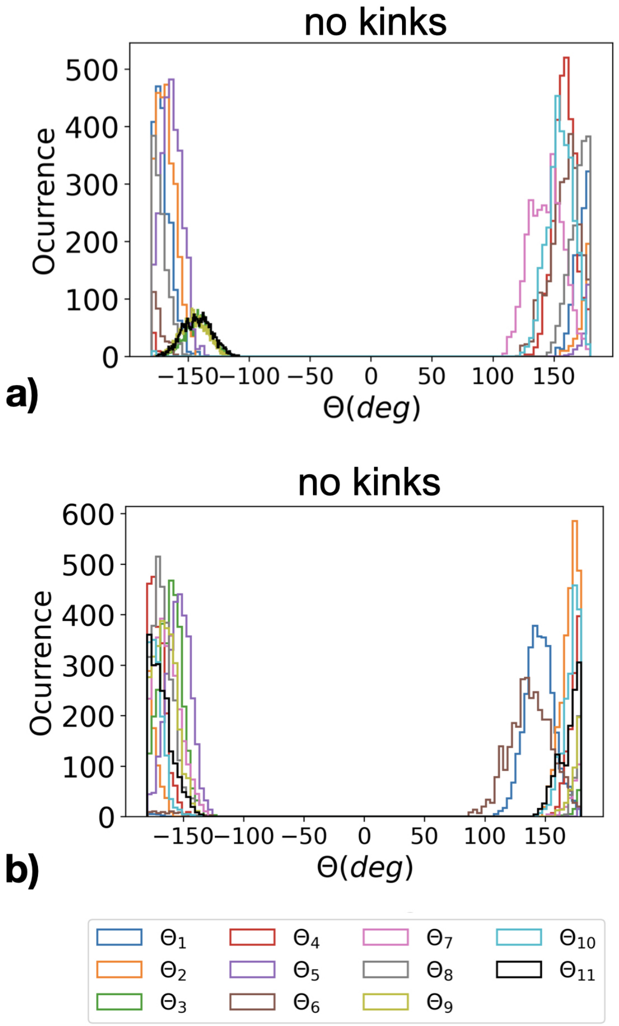


Figure S8: Distribution of dihedral angles along the P3HT chain for two molecules in the blend with no kinks.

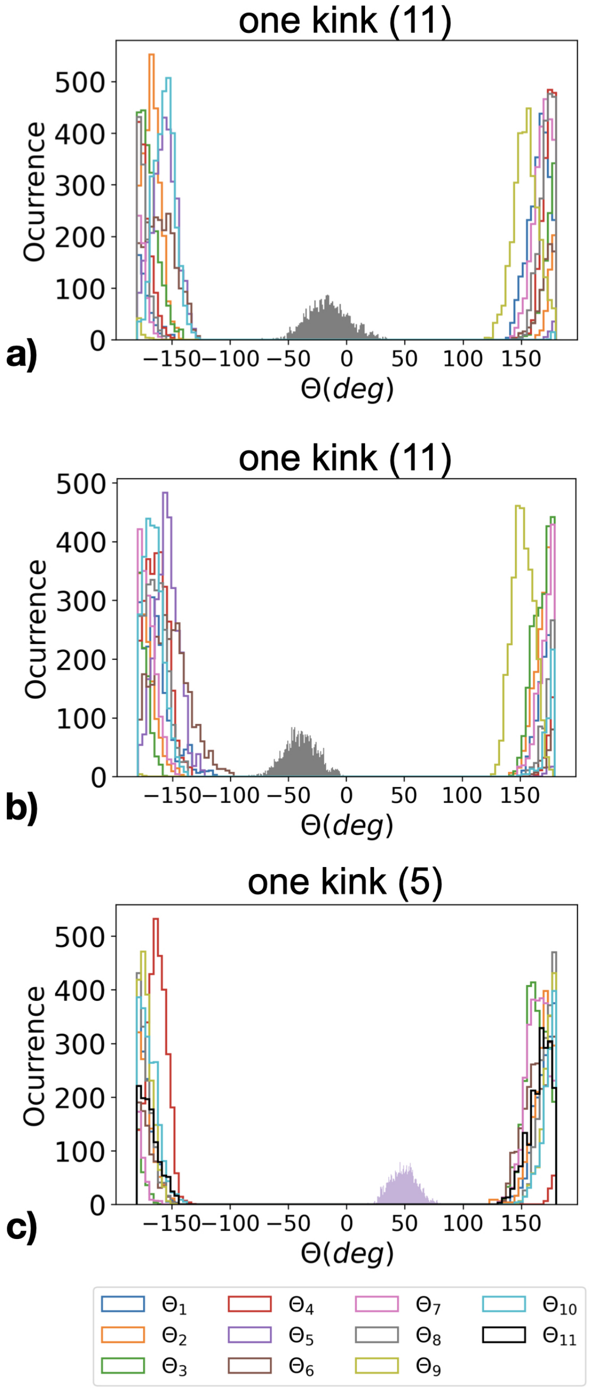


Figure S9: Distribution of dihedral angles along the P3HT chain for three molecules in the blend with one kink. Panels a) and b) correspond to two different molecules with a kink at position (11). Panel c) corresponds to a molecule with a kink at position (5).

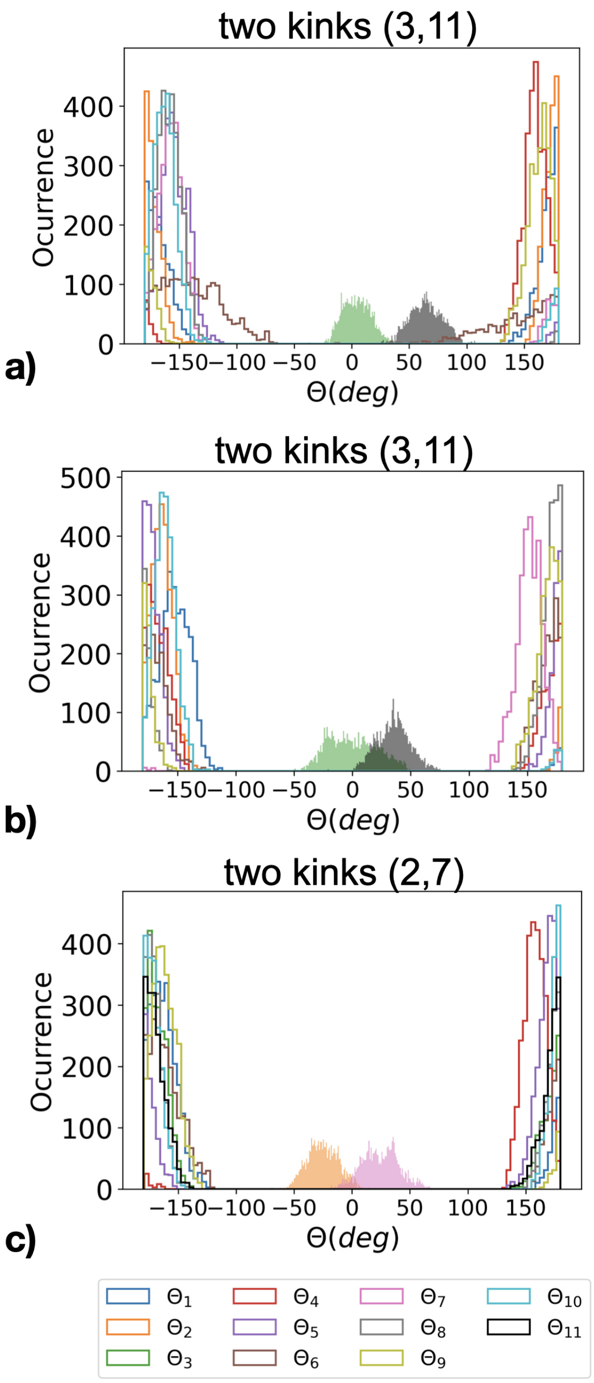


Figure S10: Distribution of dihedral angles along the P3HT chain for three molecules in the blend with two kinks. Panels a) and b) correspond to two different molecules with kinks at positions (3,11). Panel c) corresponds to a molecule with kinks at positions (2,7).

## S4 Dihedral angles trajectory from single P3HT molecules

Figure S11, S12 and S13 shows the evolution of the 11 dihedral angles in a P3HT chain along the MD trajectory, for the previously presented molecules. The blue shadow area in the figures represents the range of dihedral values in which the kink configuration exists. The molecules without kinks (Figure S11) the dihedrals angles fluctuate around a well-defined value corresponding to the planar most stable configuration of $\theta \sim 145°$. For molecules with one kink (Figure S10), it can be observed that the kink configuration and its position is maintained along the trajectory, independently on the location in the chain. A similar behaviour is reported by the data from the two-kinks molecules (Figure S13). The common pattern observed for the molecules analyzed is to maintain the kink configuration at the same position during the entire trajectory, independently on the number of kinks present in the chain. Interestingly, we observed that some molecules in the blend can experience a fast and sudden change of configuration where the dihedral angle enters the kink region for a few instants along the trajectory, which can be observed in Figure S11b around 1000 fs of the trajectory time. This behaviour roots in the local blend environment surrounding a P3HT molecule, where configurational fluctuations originated from molecule-blend interactions create short-lived kinks along the chain. The effect of bringing the molecule out of the stable planar configuration at specific chain positions will affect the exciton delocalization (as described in Section S7). Nevertheless, these configurations do not represent a substantial contribution to the configurational information of the molecule in the trajectory.

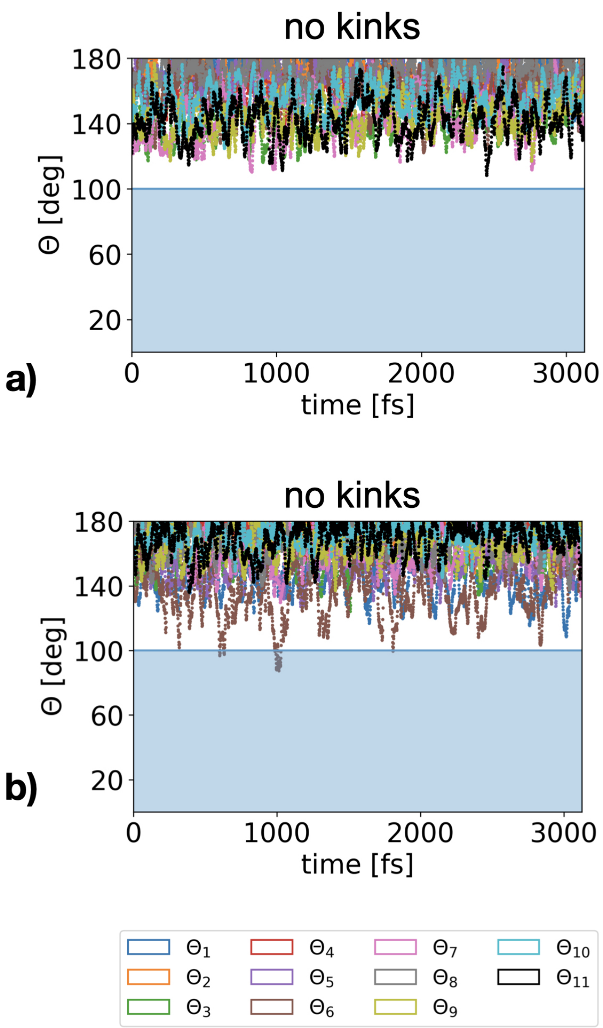


Figure S11: Dihedral angles evolution along the MD trajectory for two molecules of P3HT without kinks.

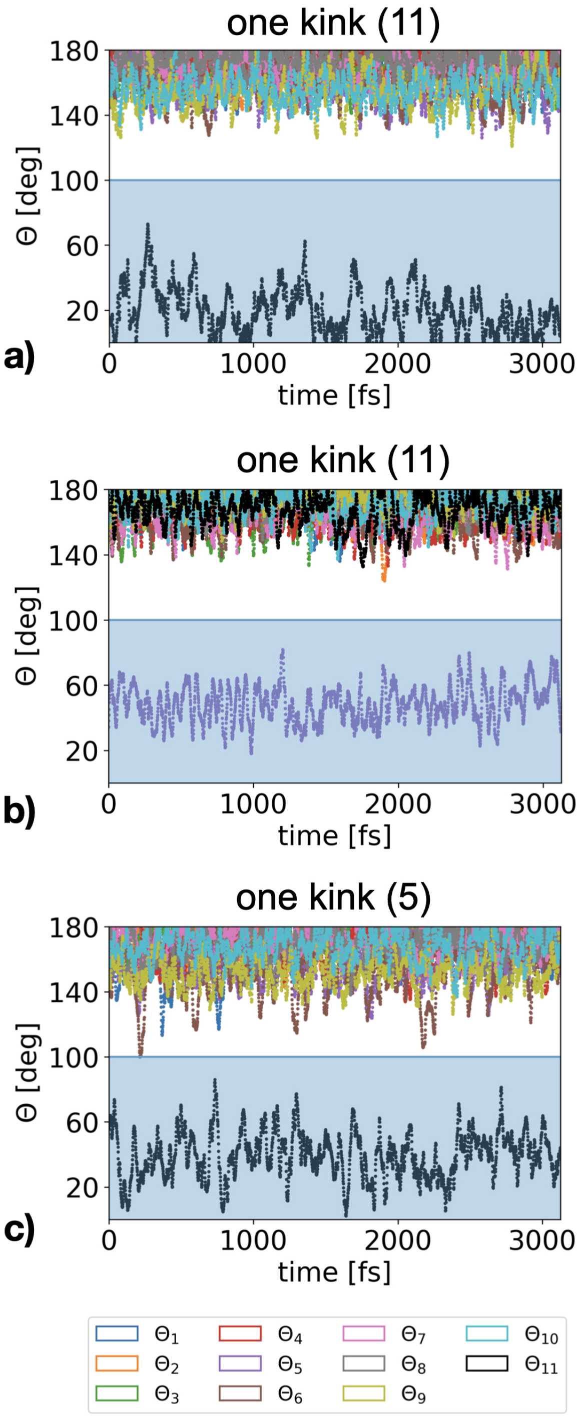


Figure S12: Dihedral angles evolution along the MD trajectory for three molecules of P3HT with one kink at position a) and b) (11) and c) (5).

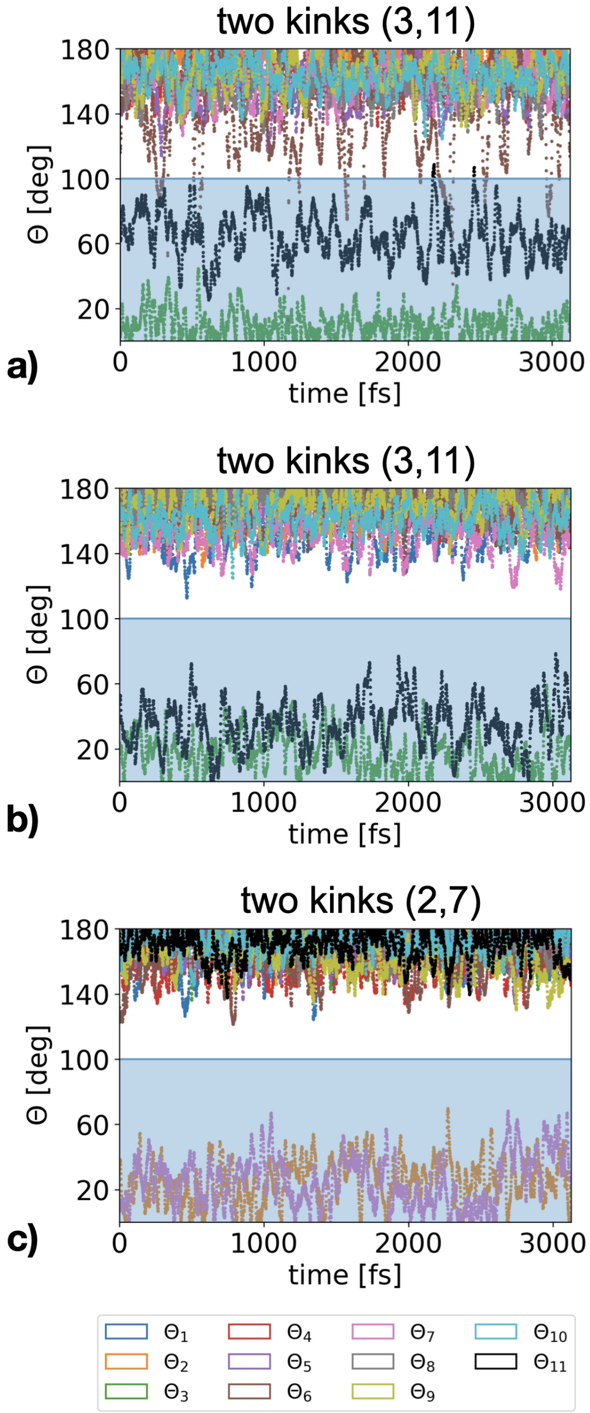


Figure S13: Dihedral angles evolution along the MD trajectory for three molecules of P3HT with two kinks at positions a) and b) (3,11) and c) (2,7).

# S5 Distribution of excitonic site-energies of single P3HT molecules

Figure S14, S15, and S16 show the distribution of excitonic site-energies obtained along the MD trajectory for the different P3HT molecules analyzed. Independently on the number of kinks in the molecule, two different energy regions can be observed, where central monomers are centered at lower energies in contrast to the end monomers which are blue-shifted by $\sim 4000$ cm$^{-1}$. This is attributed to the higher flexibility of the tiophene units at the edge of the chain.

Figure S14 reports the data for the two molecules without kinks previously presented. Panel a) reveals that the environment induces a very similar disorder for the two types of monomers (end and central positions). The data from Figure S13b corresponds to a molecule with planar configuration that for a few instants during the trajectory experiences a sudden change of configuration at position 6 and creating a short-lived kink (discussed in Section S5, Figure 10 b)). This phenomena impacts the degree of disorder of the site-energies for the monomers in the chain connected by the kink, thus broadening the distribution. In particular, in this case, the central monomers connected by this new kink at dihedral position 6 (brown and pink color histograms from Figure S14) experience a broader energy distribution compared to the rest of monomers along the chain. Figure S15 shows the distribution of site energies for three different molecules with one kink at different chain positions (specified in parenthesis). The energy distributions for the monomers connected the kink is shown with shadow histograms. A significantly broader distribution is observed for the monomers connected by the kink, that is monomer 11 for panels a) and b) and monomers 6 and 7 for panel c), in contrast to the rest of monomers central in the chain. Figure S16 corresponds to the data for the three molecules with two kinks analyzed in this work, where an analogous behaviour to the one reported for Figure S15 can be observed. The position of the kink determines the monomers in the chain experiencing a significantly

broader distribution of site-energies compared to the rest of monomers. That is for positions (3,11) (green, red, black histograms) in a) and b) and (2,7) (orange, green, pink, grey histograms) in c).

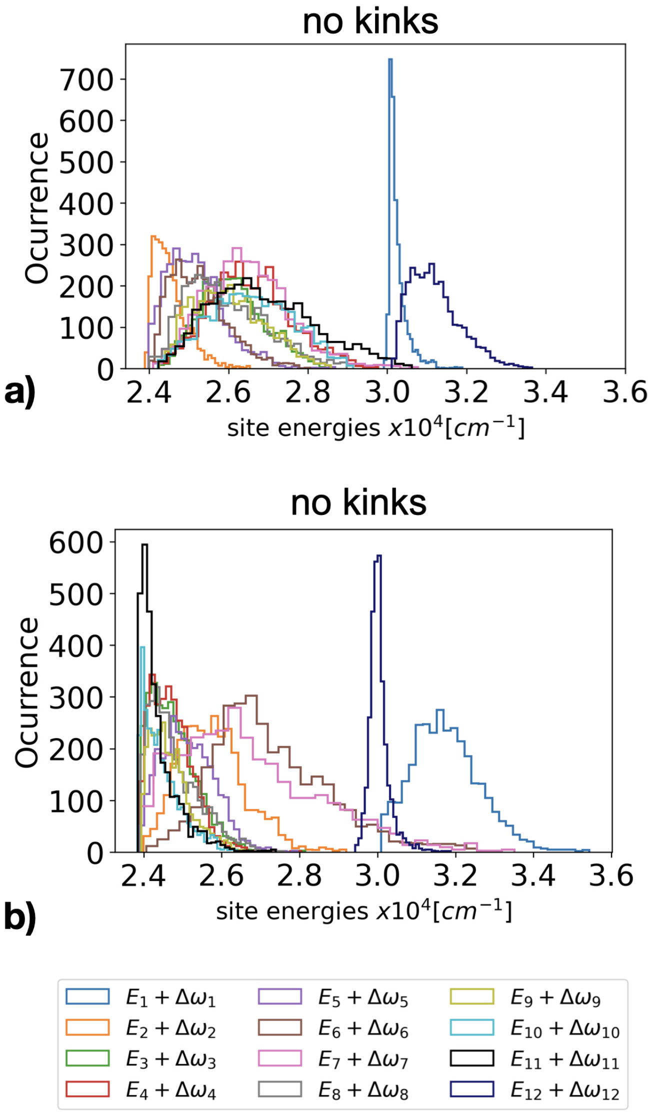


Figure S14: Distribution of monomer site-energies along the MD trajectory for two molecules of P3HT without kinks.

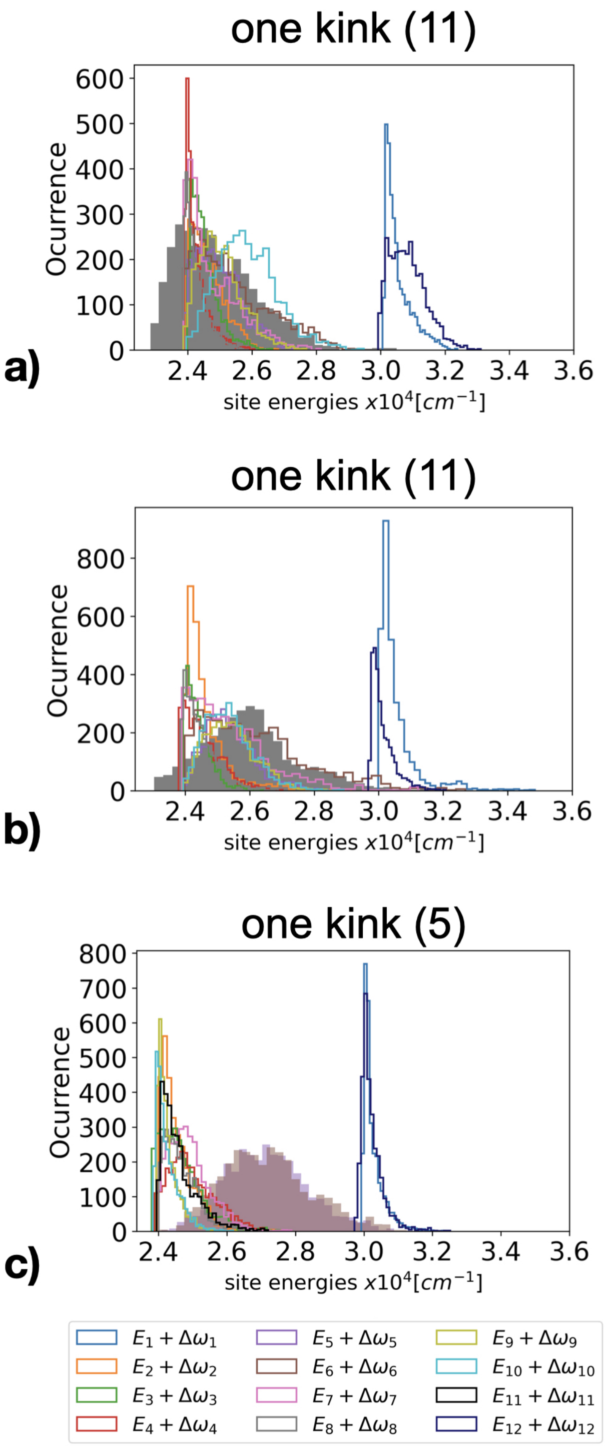


Figure S15: Distribution of monomer site-energies along the MD trajectory for two molecules of P3HT with one kink at position a) and b) (11) and c) (5).

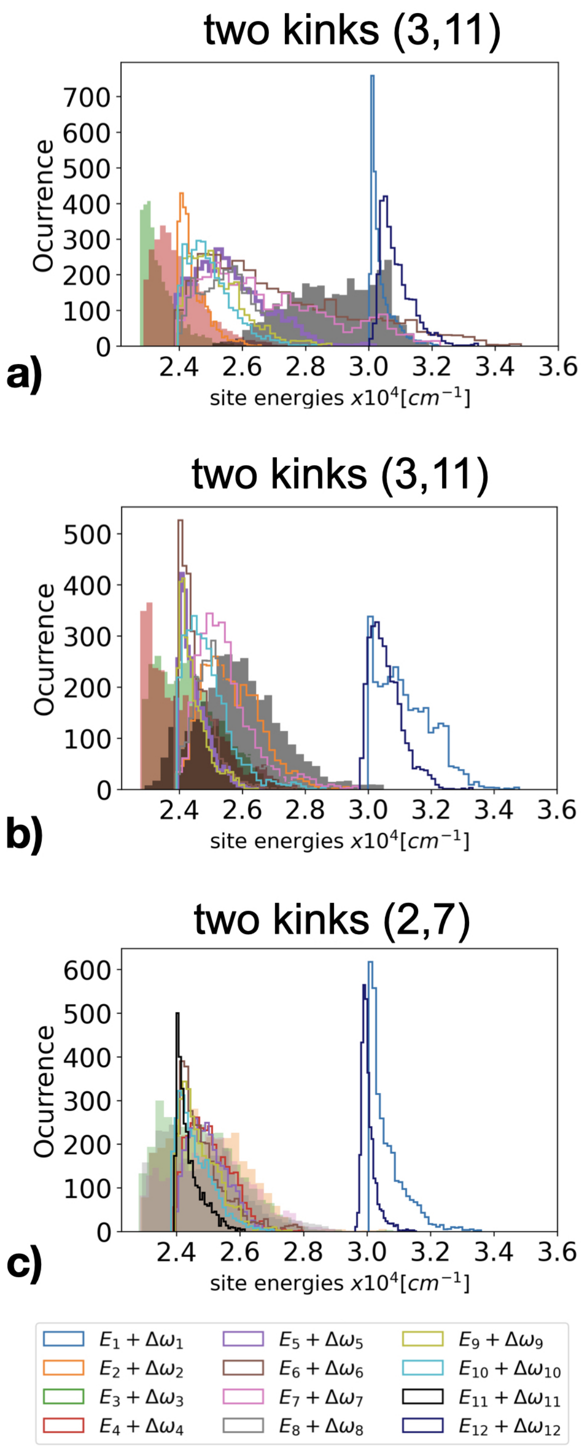


Figure S16: Distribution of monomer site-energies along the MD trajectory for two molecules of P3HT with two kinks at positions a) and b) (3,11) and c) (2,7)

# S6 Distribution of excitonic couplings of single P3HT molecules

The presence of kinks in a P3HT molecule also determines the distribution of excitonic couplings between neighbour monomers in the chain. Figures S16, S17, and S18 show the effect of the presence of kinks on the distribution of the excitonic couplings between pairs of neighbour monomers for the set of P3HT molecules previously discussed. Primarily, the presence of kinks has an opposite effect to the one reported on the excitonic energies in Section S5. For the two molecules without kinks presented (Figure S17), the disorder on the coupling is determined by the solvent shift (diagonal disorder) and small fluctuations of dihedrals along the MD trajectory. As reported in Section S5, the ten central monomers will show a similar distribution, distinct to the two end monomers in the chain. The latter is independent on the number of kinks in the chain. The presence of a kink in the chain (Figure S18) narrows the distribution of the coupling between the monomers connected by the kink, that is, coupling $J_{11,12}$ in a) and b) and couplings $J_{4,5}$, $J_{5,6}$ in c). An analogous behaviour is observed for the data with two kinks (Figure S19). The origin of this effect can be attributed to the profile of the excitonic coupling obtained from the TDDFT calculations (Figure 1 a) in the main paper), where for a kink dihedral configuration the excitonic coupling does not change significantly. Consequently, when the dihedral angle between two neighbour monomers in the chain adopts a kink configuration, the coupling between those units is more robust to the environment fluctuations.

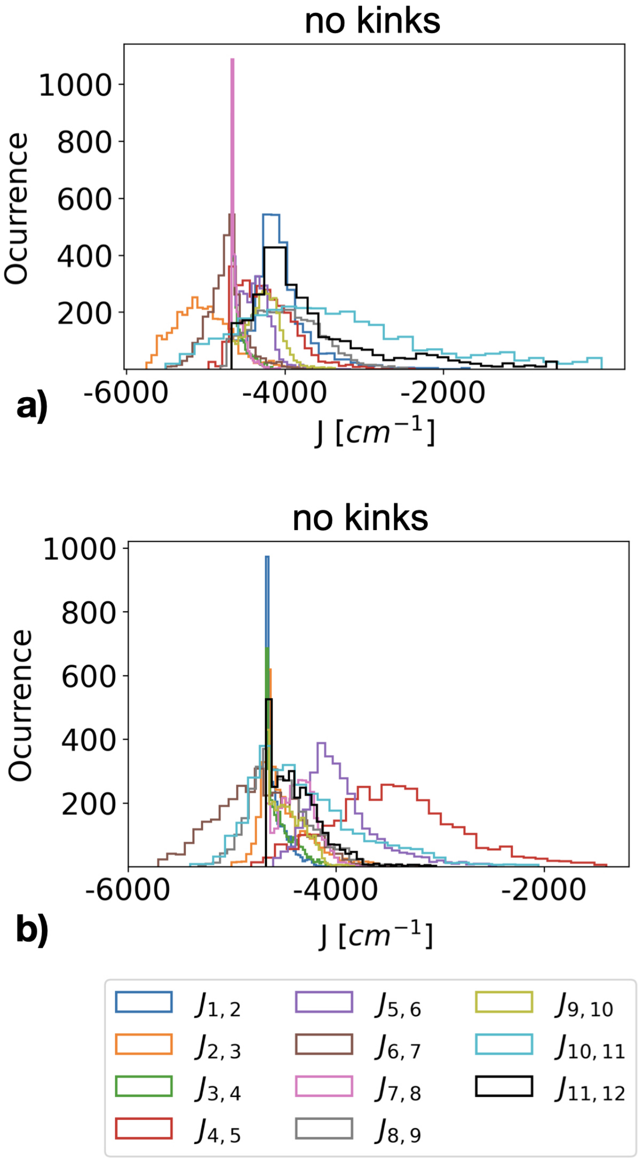


Figure S17: Distribution along the MD trajectory of excitonic couplings between pairs of neighbouring units for two molecules without kinks.

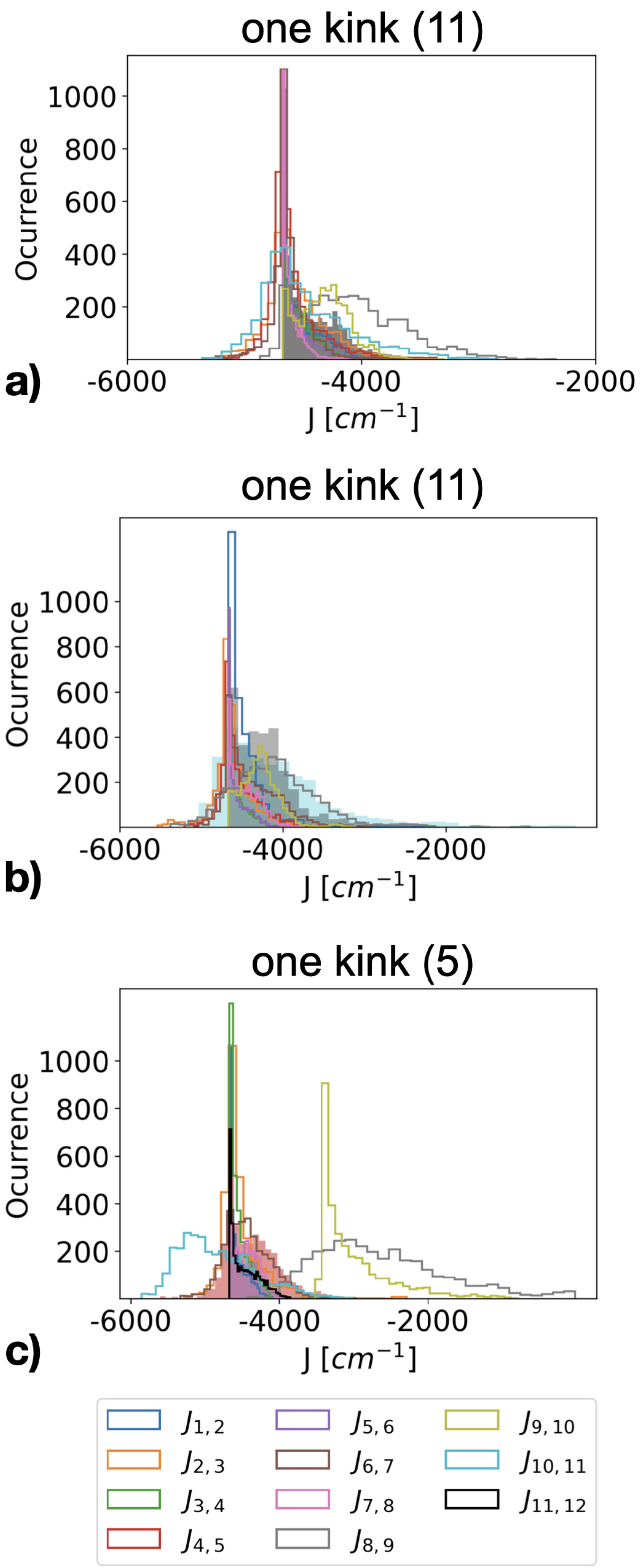


Figure S18: Distribution along the MD trajectory of excitonic couplings between pairs of neighbouring units for three molecules with one kink at position a) and b) (11) and c) (5).

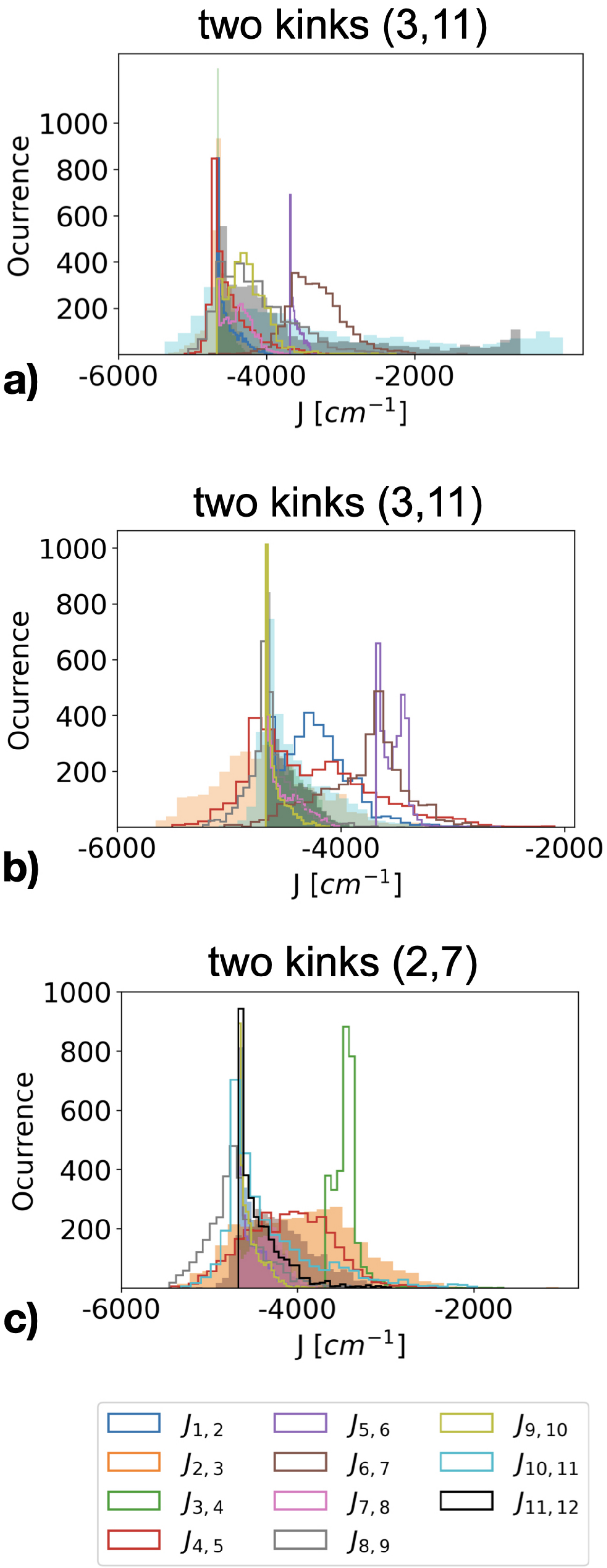


Figure S19: Distribution along the MD trajectory of excitonic couplings between pairs of neighbouring units for three molecules with two kinks at position a) and b) (3,11) and c) (2,7).

## S7 Density-matrix delocalization maps

To understand how the presence of kinks affects the exciton delocalization along the P3HT chains, we simulated the average density-matrix delocalization maps in the site representation over the trajectory time for molecules with different number of kinks in the blend. These maps, shown in Figures S19, S20 and S21, display a two-dimensional view of the exciton wavefunction on the twelve site monomers. The diagonal shows the exciton population along the different sites, and permits to extract information about the degree of delocalization along the chain. The off-diagonal elements carry information about the exciton correlation between different sites in the chain and reflect the extent of the delocalization. Overall the different maps reported show a high level of correlation between the different sites in the polymer, revealing the robustness of the coherence existing between sites. Since the maps represent the density matrix for a single molecule of P3HT and not an ensemble average, they show a high extent of correlation between the sites. As it can be extracted from the analysis of these maps, the kinks in the chain localize the exciton splitting the delocalization at the kink position.

Figure S20 shows maps for the two molecules without kinks and represents a scenario where the exciton is distributed along the chain. In contrast to panel a), panel b) shows the exciton delocalization is broken at the middle position of the chain (5). This can be ascribed to the presence of a short-lived kink at position (5), as previously discussed in the analysis of dihedrals trajectory from Figure S11b in Section S4. Panel b) shows two regions of the chain about the same length in which the exciton is localized. This configuration, despite of being statistically not representative for the geometry along the trajectory, strongly determines the exciton delocalization along the chain.

Figure S21 shows the delocalization maps for the three molecules with one kink considered in our analysis. The position of the kink (given in parenthesis) determines the point where the delocalization along the chain is broken. Panels a) and b) show a similar pattern, where most of the exciton is delocalized along most of the chain length. The molecule presenting

during the trajectory a kink at position (5) shows a localization pattern that reflects the exciton being localized on the two halves of the chain. Differences between panels a) and b) are due to fluctuations along the trajectory due to the interaction with the blend.

Figure S22 shows three different scenarios for molecules with two kinks. With two kinks, there are two nodes in the delocalization maps, splitting the delocalization along the chain in three distinct regions determined by the position of the kinks. Panels a) and b) show the exciton localized between the kinks at positions (3,11). Differences between panels a) and b) are due to fluctuations along the trajectory due to the interaction with the blend. Panel c) shows the kink at position (2) localizes the exciton mostly along the rest of the chain.

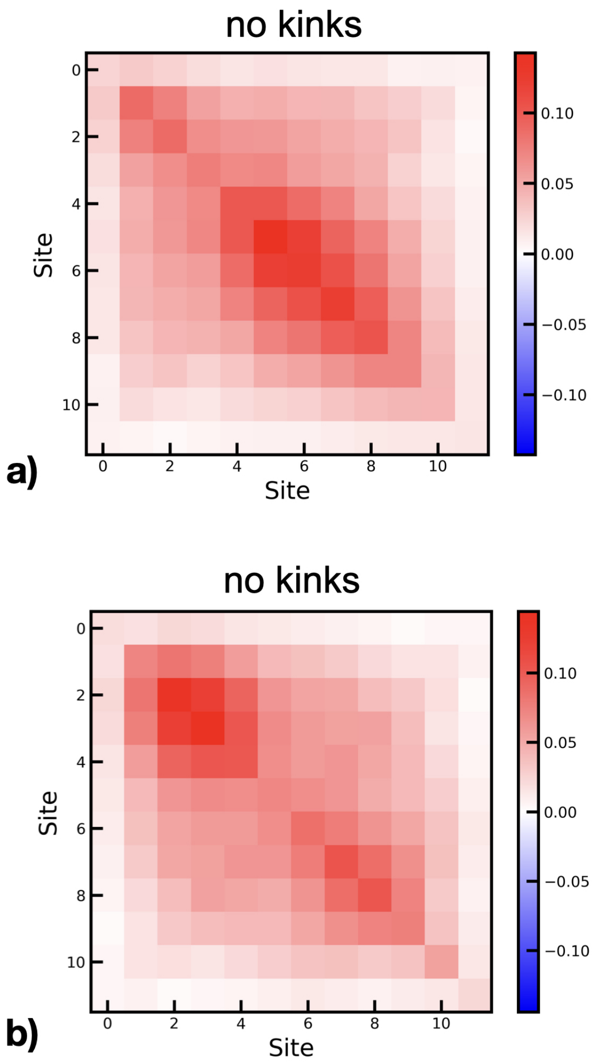


Figure S20: Density-matrix delocalization maps for two molecules without kinks.

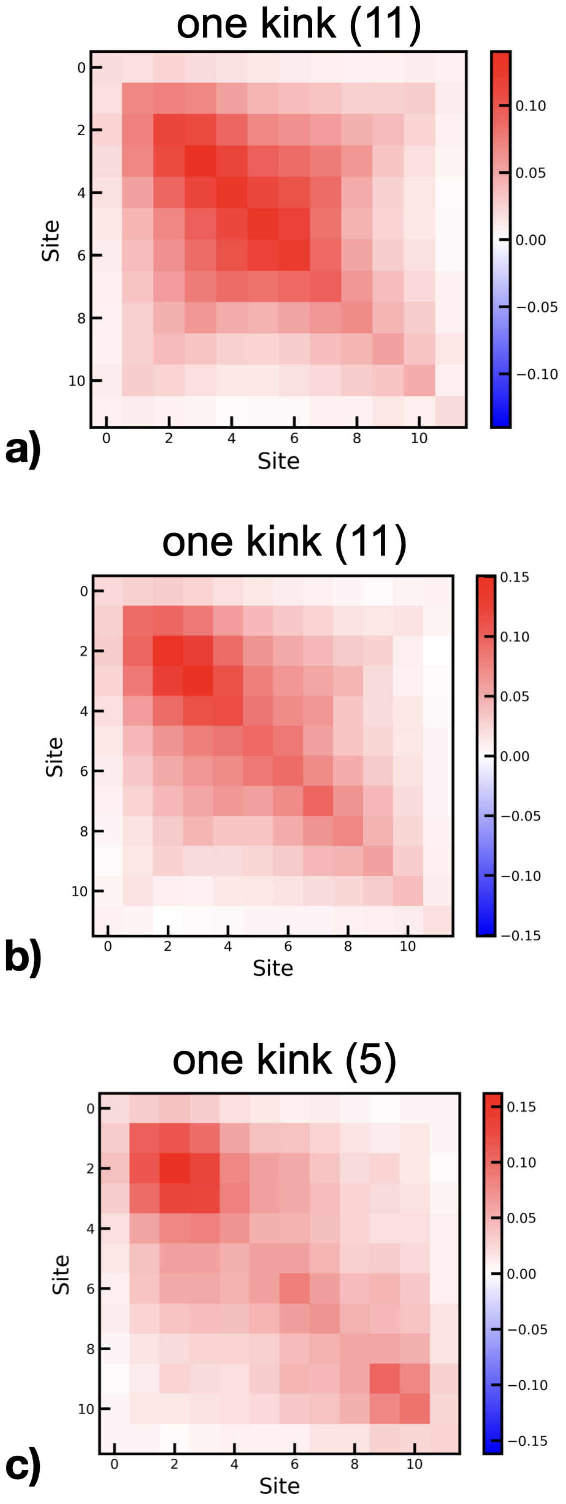


Figure S21: Density-matrix delocalization maps for three molecules with one kink at positions a) and b) (11) and c) (5).

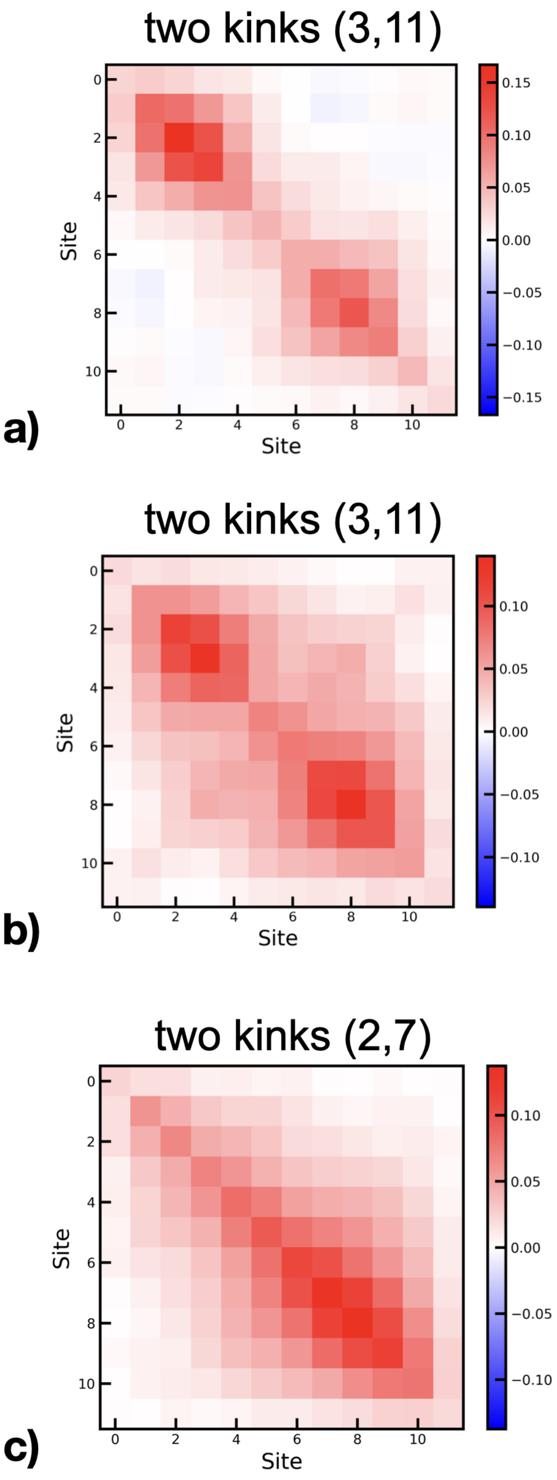


Figure S22: Density-matrix delocalization maps for three molecules with two kinks at positions a) and b) (3,11) and c) (2,7).

A quantitative analysis of the exciton delocalization in the P3HT chains is presented through the inverse participation ratio, R, which allows to estimate the distribution of the exciton delocalization lengths. The inverse participation ratio for the different P3HT molecules analyzed in the main text is included in Table S6, corresponding to an average over the trajectory. An average inverse participation ratio of 6-7 reveals a small effect of the number of kinks present in the chain. This is an evidence of the flexibility of the kinks due to the interaction with the environment, which appears to be very fluctuating for a mixture of PCBM and P3HT units. As a consequence, the effective length of the chain determined by the presence of defects (kinks) varies slightly with the presence of kinks for our simulation box. Nevertheless, a decreasing trend in $R$ is observed with the increasing number of kinks along a P3HT chain.

Table S6: Inverse participation ratio, R, for the different P3HT molecules analyzed. The left column refers to the figures presented in this section.

| molecule | inverse participation ratio, R |
|---|---|
| S16a | 6.79 |
| S16b | 6.32 |
| S17a | 7.02 |
| S17b | 6.98 |
| S17c | 5.89 |
| S18a | 5.61 |
| S18b | 6.39 |
| S18c | 6.66 |

# S8 Simulated linear absorption signals of single P3HT molecules

Figures S22, S23, and S24 show the simulated linear absorption (LA) spectra for the molecules with different number of kinks previously presented. The spectra for the two molecules without kinks is presented in Figure S23, which displays an absorption of highest intensity

centered around $\sim$ 18500 cm$^{-1}$. For both molecules, the width of the spectra is similar and $\sim$ 3000 cm$^{-1}$. The frequency of the absorption maximum does not change when increasing the number of kinks to one, as shown in Figure S24. However, the width of the spectra slightly increases to $\sim$ 4000 cm$^{-1}$. The linear absorption spectra for the analyzed molecules with two kinks (Figure S25) revealed that increasing the kinks in the chain also does not chain the position of the maximum intensity, but the width of the spectra increases to $\sim$ 5000 cm$^{-1}$, almost doubling the one found in the molecules without kinks.

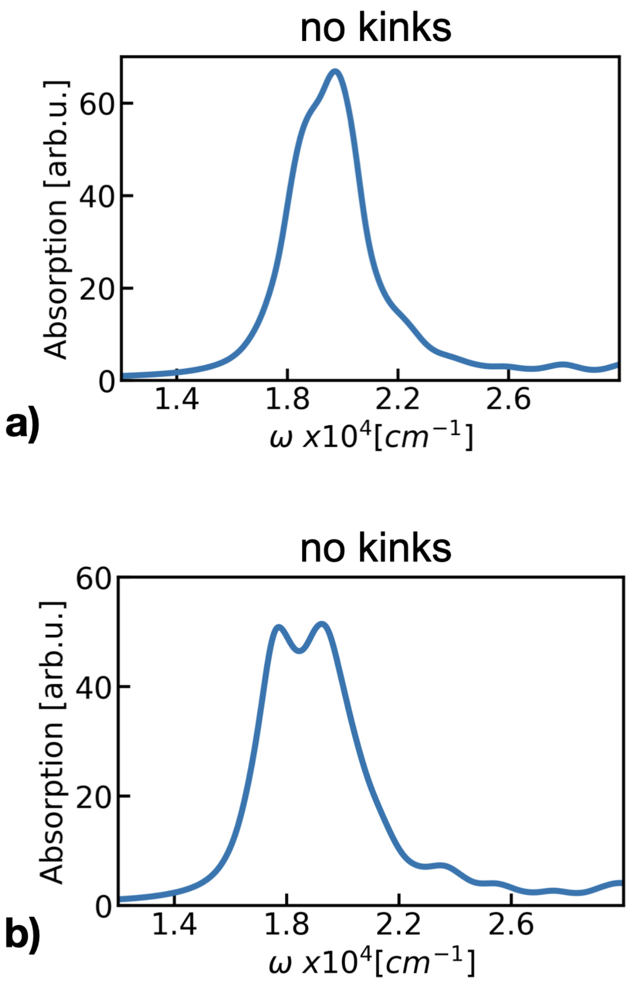


Figure S23: Simulated linear absorption spectra for two molecules without kinks.

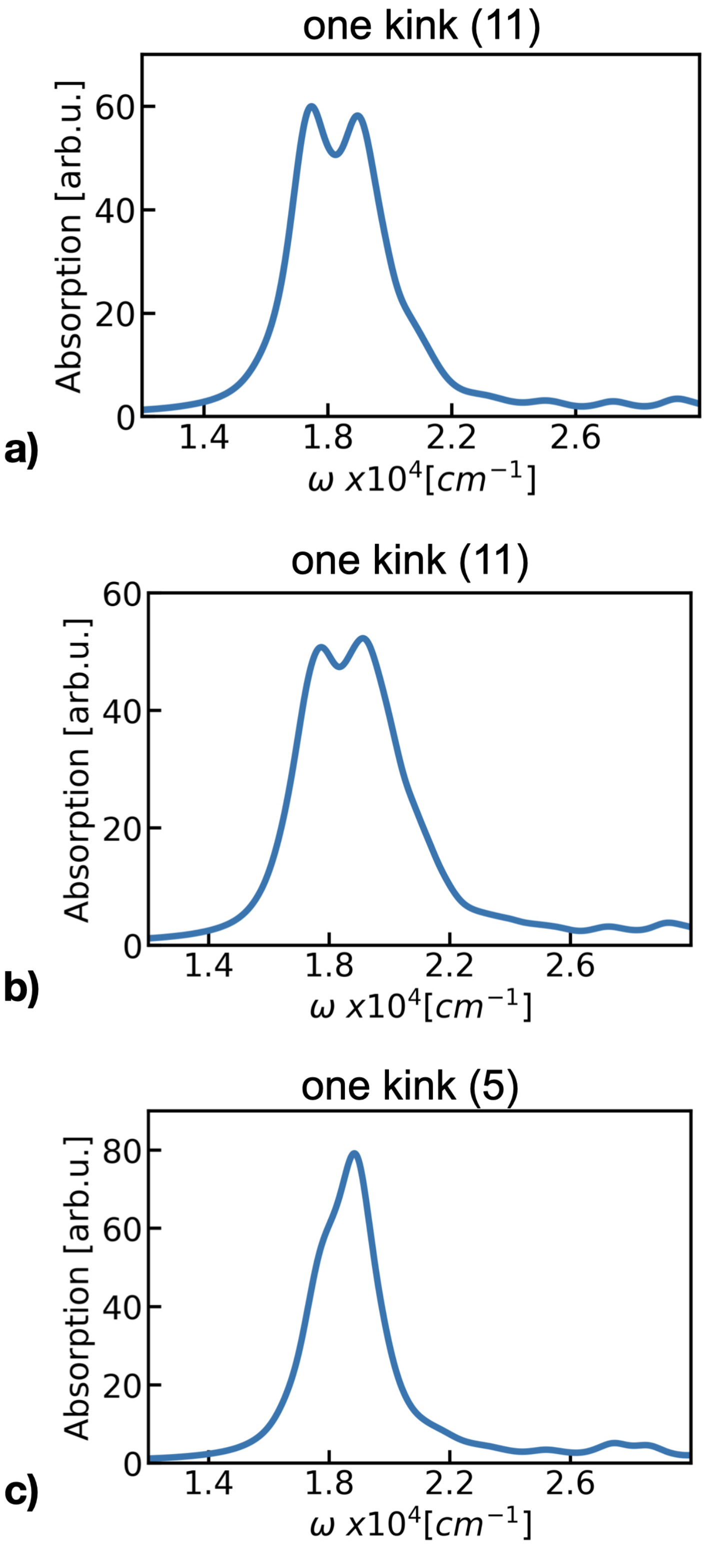


Figure S24: Simulated linear absorption spectra for three molecules with one kink at positions a) and b) (11) and c) (5).

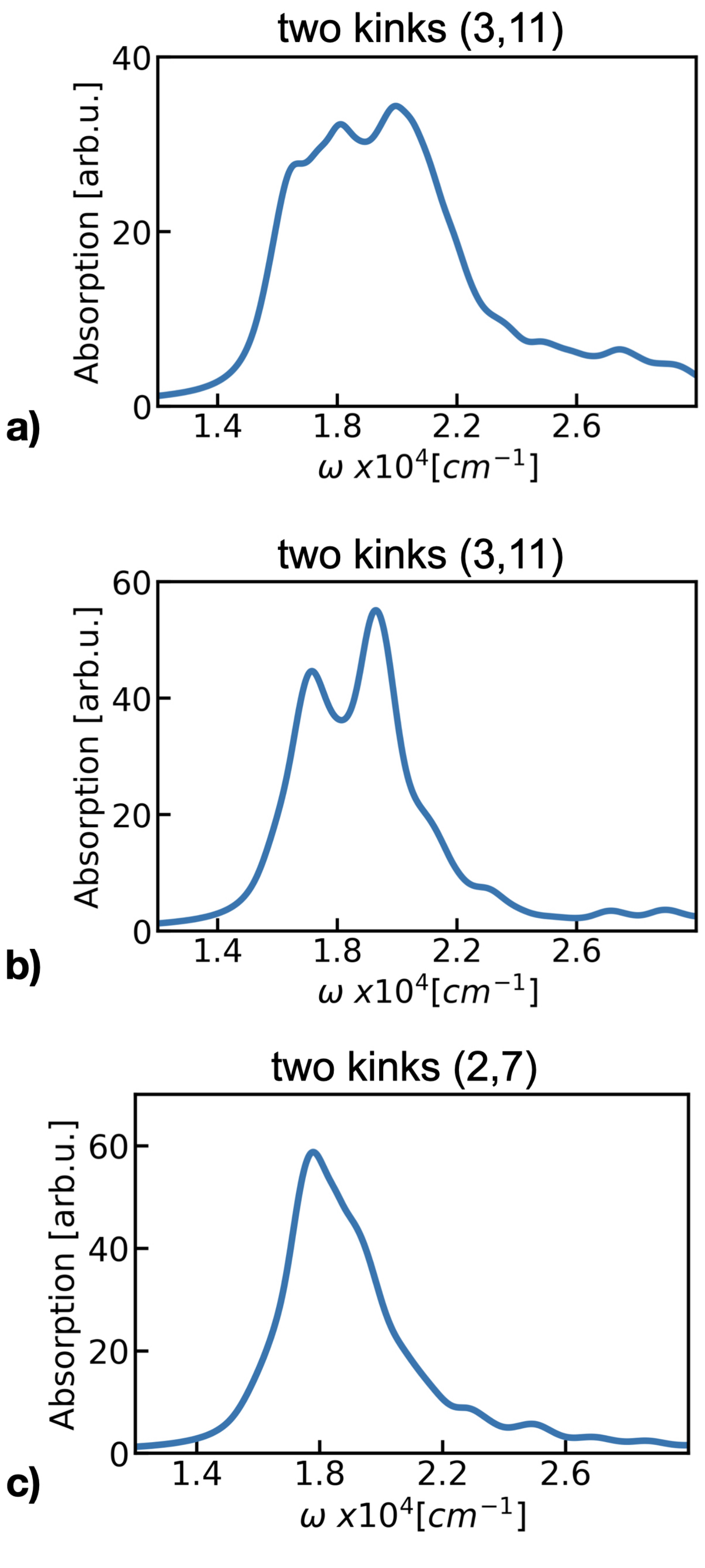


Figure S25: Simulated linear absorption spectra for three molecules with two kinks at positions a) and b) (3,11) and c) (2,7).

## S9 Comparison of the simulated linear absorption spectra of two P3HT chain lengths

In the present section we analyze the effect of chain length on the linear absorption spectra of P3HT. The blue line in Figure S26 shows the simulated spectrum of a 12-mer P3HT molecule taken from our MD simulations. The solid orange line represents the spectrum of the two 6-mer systems obtained from the two independent halves of the original 12-mer P3HT molecule after artificially setting the coupling between the two central monomers to zero. Both data contain the 1.1 eV correction factor to the adiabatic energies reported in Table S4 of Section S2. In this way the orange solid line would reproduce the scenario of two decoupled 6-mer units in the 12-mer molecule, highlighting the role of the nearest-neighbour coupling. The main absorption feature from the 6-mer + 6-mer' system broadens and shifts slightly with respect to the 12-mer and an additional band appears on the blue region, as expected for shorter conjugation lengths and in agreement with the work of Farouil et al.[2]

The dashed orange line represents the effect of a shorter polymer length of 6 units on the absorption spectrum and contains the 0.71 eV correction factor from our simulations on the 6-mer system, reported in Table S4 of Section S2. Compared to the 12-mer, a 6-mer chain shifts considerably the center of the spectrum to higher-frequencies, as depicted by the orange dashed line, in agreement with available experimental and theoretical data on pure P3HT thin-films.[2,6] This analysis reinforces our argument, discussed in the main text, where the blue-shift originated from a shorter polymer chain (dashed orange line) is stronger than the one due to a broken conjugation in the middle of a longer chain (solid orange line). This effect translates into broader signals when averaging over an ensemble of molecules with different chain lengths, as occurs in experiments. Since our MD simulation box considers P3HT units of the same length, the predicted signals are narrower in comparison with the one reported by the experiments.[2,6] Besides, it should be borne in mind that in our simulations we mimic the physical environment created from a P3HT:PCBM blend, whereas the experimental

data reported[2,6] describes the behaviour of a pure P3HT sample. Due to these differences a rigorous comparison cannot be made.

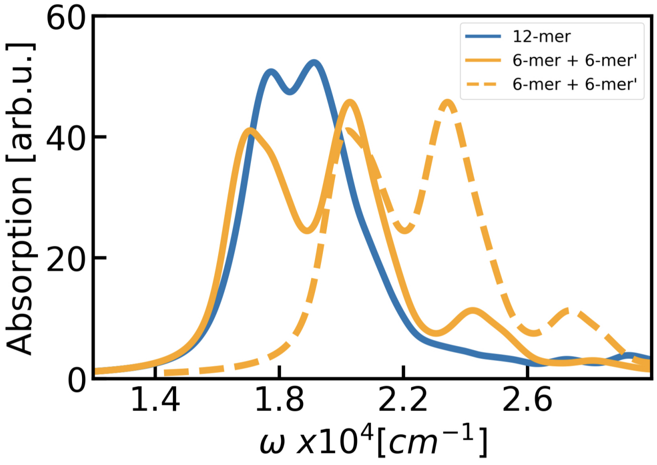


Figure S26: Simulated spectrum of a 12-mer P3HT molecule (blue), spectrum obtained from the two independent halves of the original 12-mer P3HT molecule after artificially setting the coupling between the two central monomers to zero (orange solid line) and spectrum of a 6-mer molecule (dashed line). See text for more details.

## S10 Simulated 2DES signals of single P3HT molecules

Figures S26, S27, and S28 show the simulated 2DES and a cut along the main diagonal for the different molecules analyzed. We used the parallel polarization two-dimensional electronic spectra for a waiting time equal zero. For all molecules, common features similar to the ones reported in the main paper for the ensemble weighted average 2DES spectra can be observed. This includes a negative diagonal peak originated from a ground-state bleach (GSB) and stimulated emission (SE) contributions and a positive cross-peak corresponding to an excited-state absorption (ESA) process. The GSB and SE features overlap at a diagonal frequency of $\sim 17000$ cm$^{-1}$ and relate to the central peak position of the linear absorption spectra reported. The frequency shift observed between the linear and nonlinear features is explained in the main paper. The cross-peak (ESA) appears at an excitation frequency of 16500 cm$^{-1}$ and detection frequency of 17500 cm$^{-1}$ for the different molecules presented. The cut along the diagonal of the 2DES reveals in all cases an inhomogeneous broadening of $\sim 1000$ cm$^{-1}$ originated from the single molecule contribution. Some molecules presenting two kinks give rise to a larger inhomogeneous broadening, though their contribution to the ensemble is negligible.

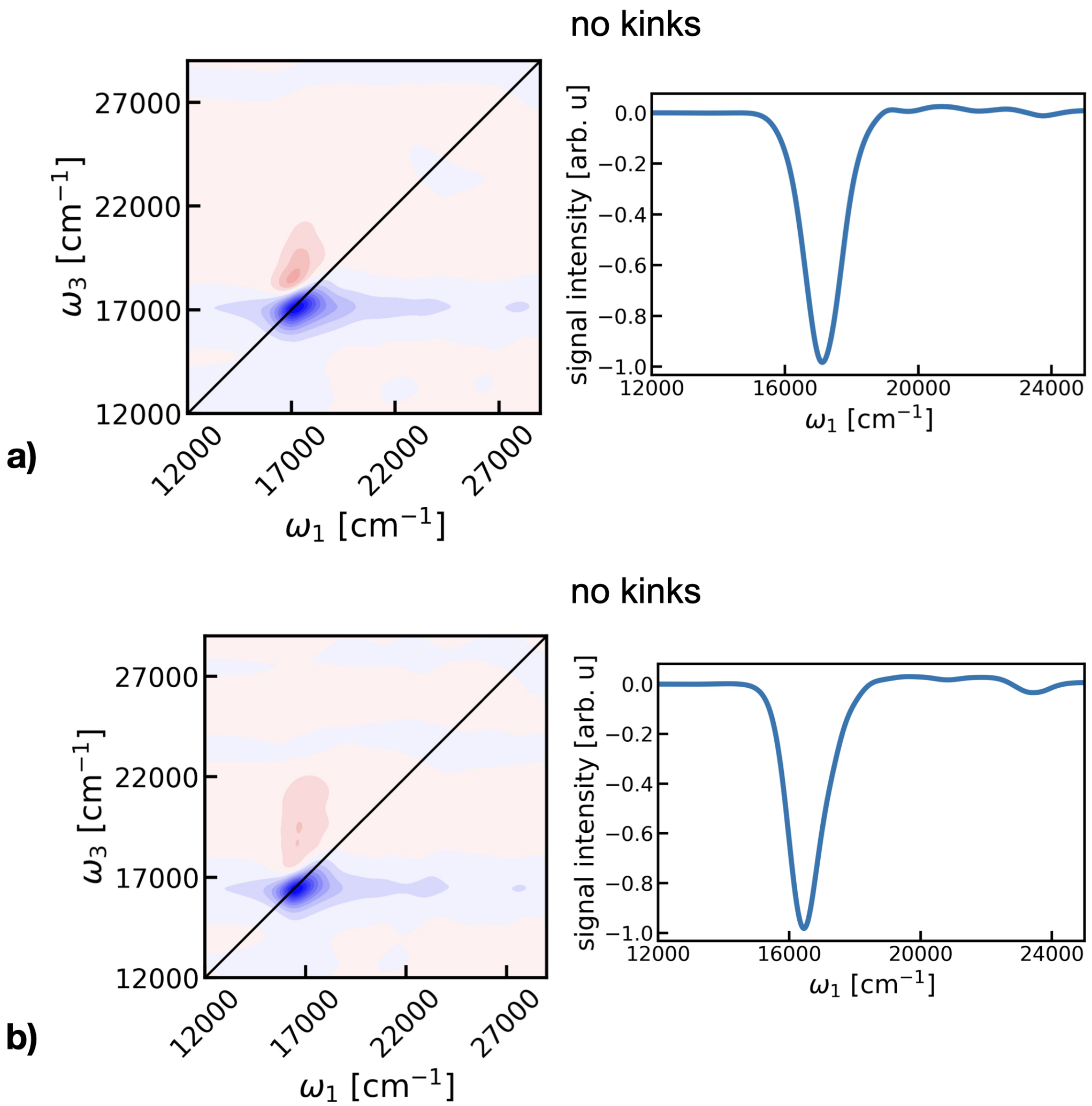


Figure S27: Simulated 2DES (left) and diagonal cut (right) for two molecules without kinks.

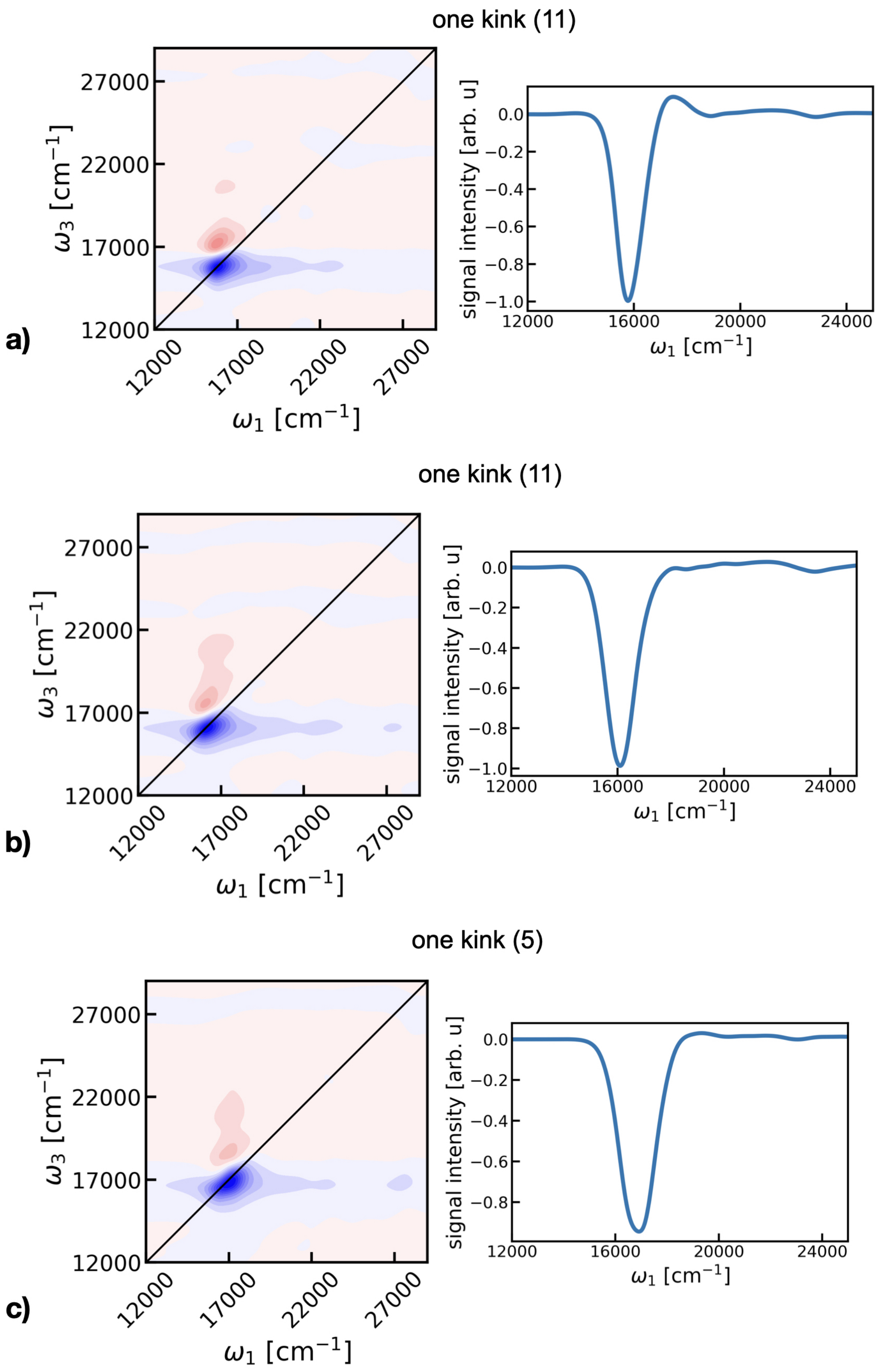


Figure S28: Simulated 2DES (left) and diagonal cut (right) for three molecules with one kink at positions a) and b) (11) and c) (5).

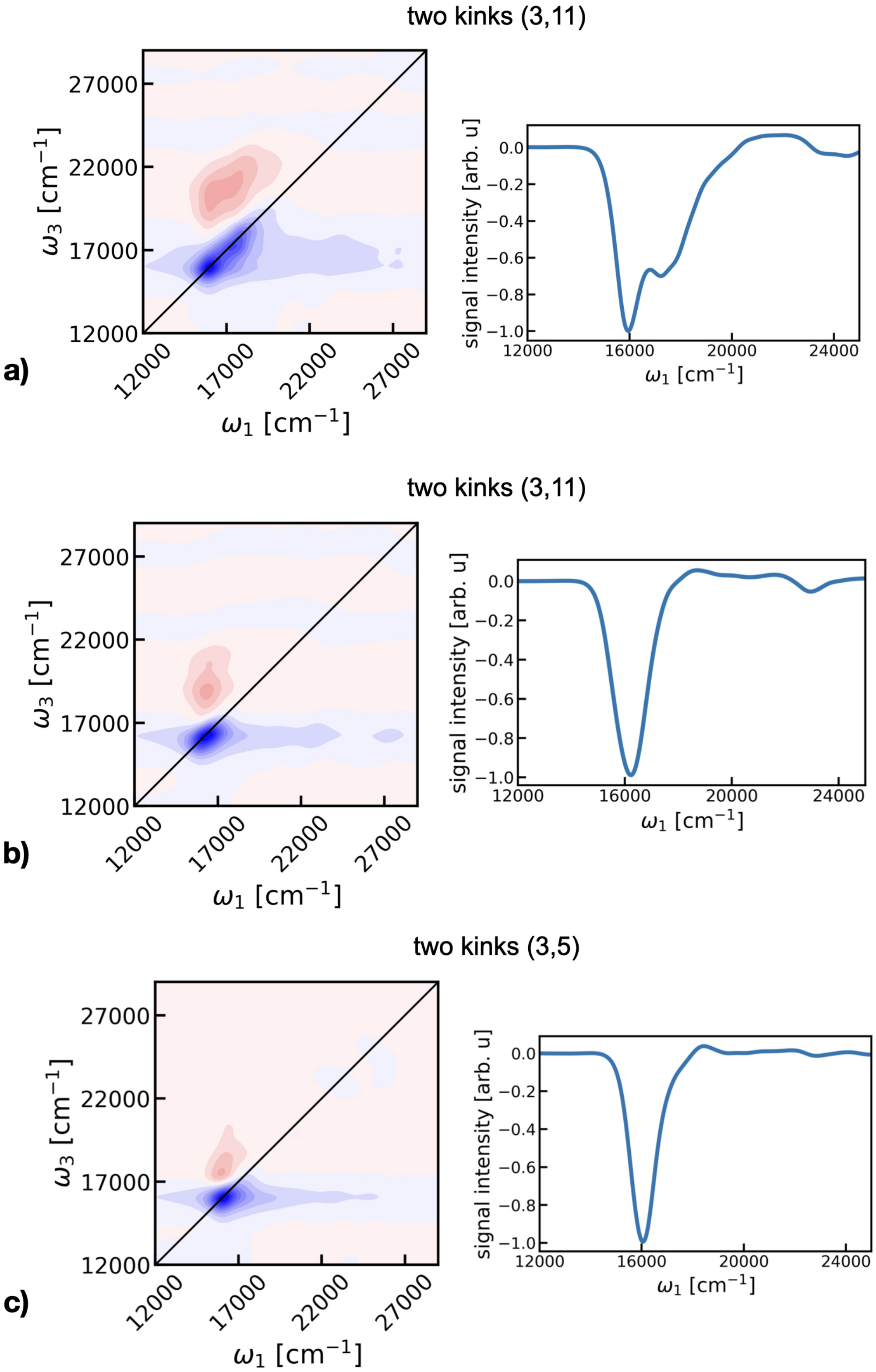


Figure S29: Simulated 2DES (left) and diagonal cut (right) for three molecules with two kinks at positions a) and b) (3,11) and c) (2,7).

Lastly, Figure S30 shows the simulated 2DES for the ensemble weighted-averaged spectra for the increasing waiting time $T_{\text{pop}}$ indicated on the top of the plots. As the waiting time in the signal increases, the initial correlation observed in the spectral features shown at zero waiting time is lost due to the effect of the fluctuating environment surrounding the single molecule. From this analysis, an homogeneous dephasing time relating to the electronic dephasing of 100 fs in the individual molecules is estimated.

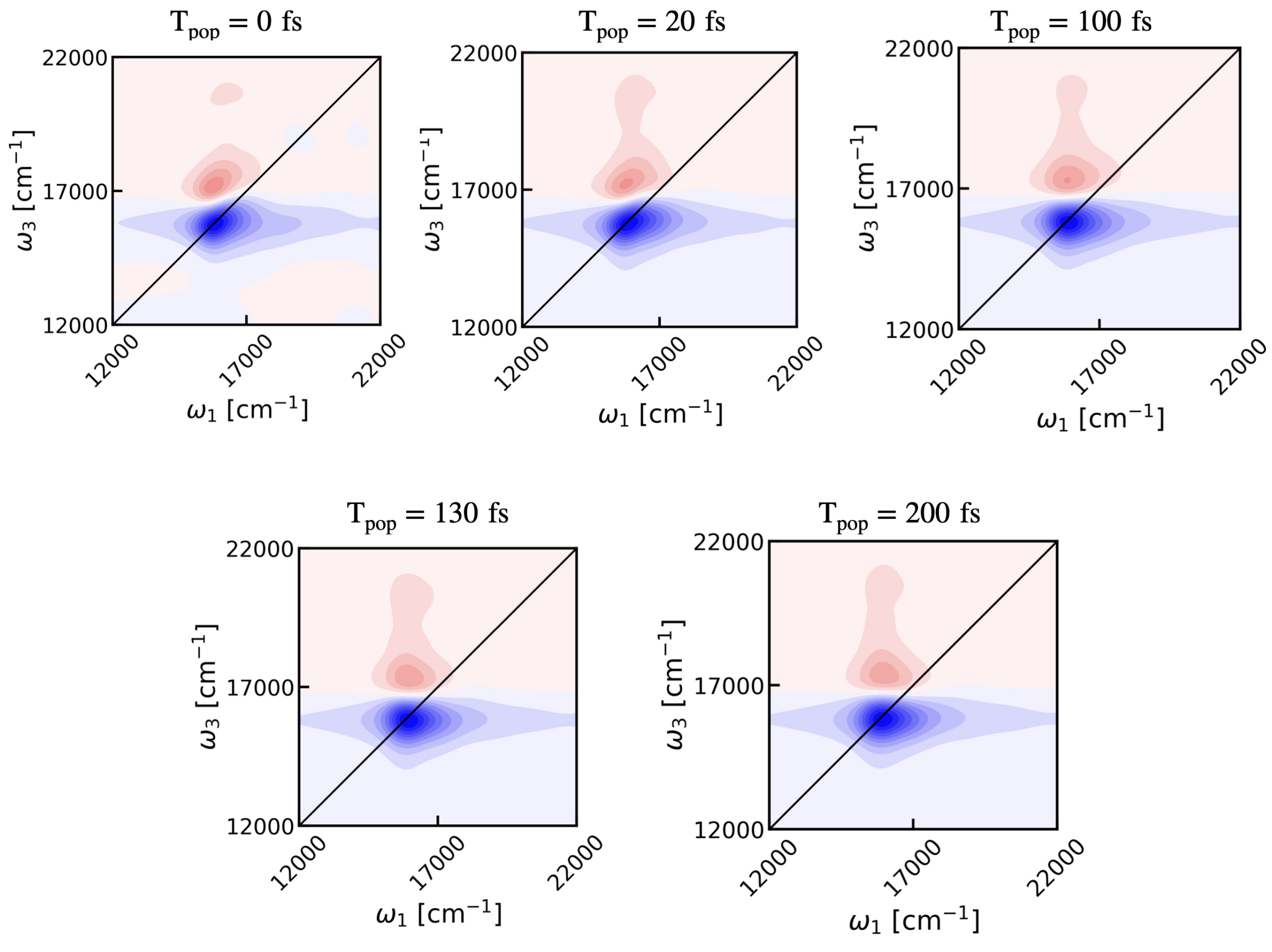


Figure S30: Simulated ensemble weighted-averaged 2DES for increasing waiting times $T_{\text{pop}}$.

# References


(1) Alessandri, R.; Uusitalo, J.; de Vries, A.; Havenith, R.; Marrink, S. Bulk Heterojunction Morphologies with Atomistic Resolution from Coarse-Grain Solvent Evaporation Simulations. *Journal of the American Chemical Society* **2017**, *139*, 3697-3705.

(2) Farouil, L.; Alary, F.; Bedel-Pereira, E.; Heully, J.-L. Revisiting the Vibrational and Optical Properties of P3HT: A Combined Experimental and Theoretical Study. *The Journal of Physical Chemistry A* **2018**, *122*, 6532–6545.

(3) Neese, F. The ORCA Program System. *Wiley Interdisciplinary Reviews: Computational Molecular Science* **2012**, *2*, 73-78.

(4) Plasser, F. TheoDORE: a Toolbox for a Detailed and Automated Analysis of Electronic Excited State Computations. *J. Chem. Phys.* **2020**, *152*, 084108.

(5) Rathnachalam, S.; Menger, M.; Faraji, S. Influence of the Environment on Shaping the Absorption of Monomeric Infrared Fluorescent Proteins. *J. Phys. Chem. B* **2021**, *125*, 2231–2240.

(6) Thiessen, A.; Vogelsang, J.; Adachi, T.; Steiner, F.; Bout, D. V.; Lupton, J. M. Unraveling the Chromophoric Disorder of Poly(3-hexylthiophene). *PNAS* **2013**, *110*, E3550–E3556.
